# Associations between Serum Mineral Nutrients, Gut Microbiota, and Risk of Neurological, Psychiatric, and Metabolic Diseases: A Comprehensive Mendelian Randomization Study

**DOI:** 10.3390/nu16020244

**Published:** 2024-01-12

**Authors:** Wang Li, Bo-Min Lv, Yuan Quan, Qiang Zhu, Hong-Yu Zhang

**Affiliations:** 1 Hubei Key Laboratory of Agricultural Bioinformatics, College of Informatics, Huazhong Agricultural University, Wuhan 430070, China; lioone9@webmail.hzau.edu.cn (W.L.); bominlv@fudan.edu.cn (B.-M.L.); quanyuan@mail.hzau.edu.cn (Y.Q.); zhy630@mail.hzau.edu.cn (H.-Y.Z.); 2 Human Phenome Institute, Fudan University, Shanghai 200438, China; 3 Key Laboratory of Smart Farming for Agricultural Animals, College of Informatics, Huazhong Agricultural University, Wuhan 430070, China

**Keywords:** mineral nutrient, gut microbiota, metabolic diseases, neurological diseases, psychiatric diseases, mendelian randomization study

## Abstract

Recent observational studies have reported associations between serum mineral nutrient levels, gut microbiota composition, and neurological, psychiatric, and metabolic diseases. However, the causal effects of mineral nutrients on gut microbiota and their causal associations with diseases remain unclear and require further investigation. This study aimed to identify the associations between serum mineral nutrients, gut microbiota, and risk of neurological, psychiatric, and metabolic diseases using Mendelian randomization (MR). We conducted an MR study using the large-scale genome-wide association study (GWAS) summary statistics of 5 serum mineral nutrients, 196 gut microbes at the phylum, order, family, and genus levels, and a variety of common neurological, psychiatric, and metabolic diseases. Initially, the independent causal associations of mineral nutrients and gut microbiota with diseases were examined by MR. Subsequently, the causal effect of mineral nutrients on gut microbiota was estimated to investigate whether specific gut microbes mediated the association between mineral nutrients and diseases. Finally, we performed sensitivity analyses to assess the robustness of the study results. After correcting for multiple testing, we identified a total of 33 causal relationships among mineral nutrients, gut microbiota, and diseases. Specifically, we found 4 causal relationships between 3 mineral nutrition traits and 3 disease traits, 15 causal associations between 14 gut microbiota traits and 6 disease traits, and 14 causal associations involving 4 mineral nutrition traits and 15 gut microbiota traits. Meanwhile, 118 suggestive associations were identified. The current study reveals multiple causal associations between serum mineral nutrients, gut microbiota, risk of neurological, psychiatric, and metabolic diseases, and potentially provides valuable insights for subsequent nutritional therapies.

## 1. Introduction

The prevalence of neurological, psychiatric, and metabolic diseases is high and on the rise globally with modernizing societies and aging populations. Neurological diseases, including Alzheimer’s disease, multiple sclerosis, stroke, and psychiatric diseases such as autism spectrum disorder and major depressive disorder, significantly contribute to the global health burden as leading causes of disability and mortality [1]. Metabolic diseases, such as type 2 diabetes and obesity, are complex and multifactorial. Epidemiological studies indicate a rise in the global prevalence of metabolic diseases from 2000 to 2019 [2]. Therefore, there is an urgent need for more effective therapeutic and preventive strategies, given the rising incidence and disability rates of neurological, psychiatric, and metabolic diseases, as well as the global trend towards their increase.

The incidence of neurological, psychiatric, and metabolic diseases is influenced by multiple factors, such as genetics, lifestyle, and environment. Recent evidence underscores the critical role of gut microbiota in host health, particularly in metabolism and immune regulation [3]. The gut microbiota and the neurological system interact bidirectionally through multiple pathways. The “brain–gut axis” refers to the bidirectional regulatory pathway between the central nervous system and the gut, with the interplay of the gut microbiota and the gut-brain axis labelled as the gut microbiota–gut–brain axis [4]. The enteric gut microbiota–gut–brain axis encompasses various pathways, including neuroanatomical pathways, the intestinal immune system, and neurotransmitters and neuromodulators synthesized by the gut microbiota. Furthermore, a growing number of studies are concentrating on the potential role of gut microbiota in the development of metabolic diseases. The gut microbiota can affect the function of the intestinal mucosal barrier and nutrient absorption, influence energy metabolism and hormone secretion, and contribute to the development of metabolic diseases through immune regulation and the inflammatory response [5,6]. In addition to gut microbiota, mineral nutrients also play a crucial role in maintaining health. The effects of some mineral elements on health may be due to their role in the structure and function of neurons, or their involvement in the body’s immune response and oxidative stress response [7,8]. Moderate intake of metallic mineral elements is important for preventing neurological, psychiatric, and metabolic diseases, as well as for maintaining good health [9,10]. Meanwhile the stability and health of the gut microbiota are influenced by multiple factors, notably mineral nutrients concentration. Mineral nutrients are essential for many enzymes and are involved in many biochemical reactions. Reduced serum mineral concentrations may affect the growth and metabolism of gut microbes. In addition, both excessive and inadequate levels of certain mineral nutrients can alter the composition of the gut microbiota, as some bacteria rely on these nutrients for growth. Therefore, serum mineral concentrations should be regarded as an important factor influencing the composition and health of the gut microbiota.

Neurological, psychiatric, and metabolic diseases possess complex aetiologies, requiring further research into their underlying causes and pathological mechanisms to enhance our understanding and treatment of these diseases. This will assist in developing new treatments and preventive measures, ultimately reducing the burden of these diseases on patients and society. Therefore, understanding the potential roles of gut microbiota and mineral nutrition is essential for maintaining their healthy balance, as well as for the prevention and treatment of neurological, psychiatric, and metabolic diseases.

Traditionally, causality has been inferred through randomized controlled trials (RCTs) aimed at assessing the causal effect of interventions on specific outcomes. However, RCTs have limitations, including potential confounding factors, reverse causality, and difficulties in implementation due to ethical constraints [11,12]. Mendelian randomization, an alternative approach, employs naturally occurring genetic variation to assess the causality of specific factors on outcomes [13]. MR exploits the random assignment of genetic alleles that affect exposure and avoids interference from unobserved confounding factors and reverse causality bias, providing advantages over other study designs [14,15].

**Figure 1:**
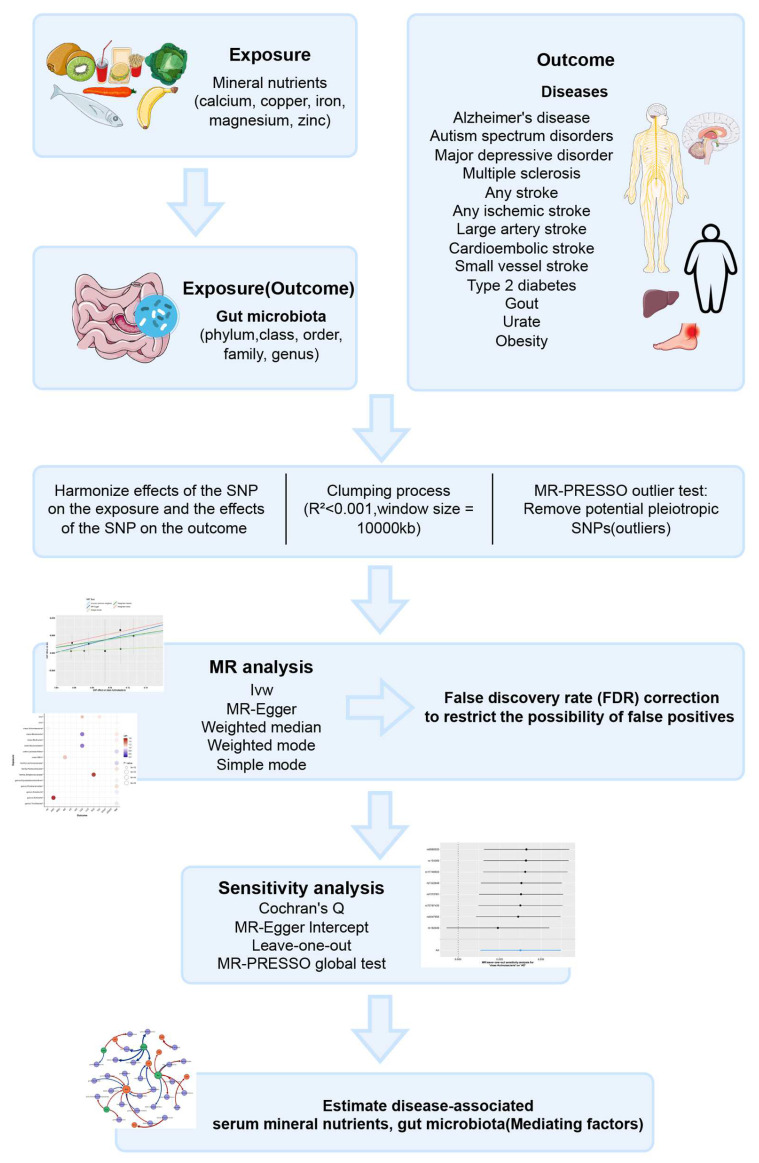
Descriptions of the overall workflow of MR analysis.

Compared to existing MR studies on the association between mineral nutrients or gut microbiota and disease, we employed MR to more systematically and comprehensively investigate the causal effects between various mineral nutrients, gut microbiota, and prevalent neurological, psychiatric, and metabolic diseases. Furthermore, we explored the associations between mineral nutrients and gut microbiota to identify gut microbes that may mediate the associations between mineral nutrients and diseases, thereby providing new ideas and methods for the prevention and treatment of related diseases. Figure 1 provides a brief overview of this process.

## 2. Methods

### 2.1. Exposure Data

Based on observational studies, we selected five serum mineral nutritional traits (calcium, copper, iron, magnesium, and zinc) as exposure factors. The data were derived from a recent large-scale Genome-Wide Association Study (GWAS) conducted on individuals of European ancestry [16,17,18,19]. Calcium, copper, iron, magnesium, and zinc are vital nutrients for the human body. Including these minerals in our research can help guide the development of dietary recommendations and supplementation strategies. These minerals, widespread in the body, perform crucial physiological functions, including neural transmission, bone health maintenance, immune support, cardiovascular wellbeing, and metabolic regulation. Investigating these minerals may reveal their associations with neurological, psychiatric, and metabolic diseases [16,17,18,19]. In addition, gut microbiota was also included as exposure in the MR analysis. The GWSA summary statistics of gut microbiota taxa were obtained from the international consortium MiBioGen. The MiBioGen consortium included genomic and gut microbiota data from 24 cohorts of more than 18,000 people of various ethnicities including Europe, America, the Middle East, and East Asia, making it the largest GWAS of gut microbiota to date [20]. After removing unknown gut microbes, a total of 196 taxa (119 genera, 32 families, 20 orders, 16 classes, and 9 phyla) were included in this study. It is worth noting that gut microbiota was also included as the outcome when investigating the association of mineral nutrients on the gut microbiota.

### 2.2. Outcome Data

We extracted GWAS summary statistics for various neurological and psychiatric diseases, including Alzheimer’s disease (AD), autism spectrum disorder (ASD), major depressive disorder (MDD), multiple sclerosis (MS), stroke (any stroke (AS), any ischemic stroke (AIS), cardioembolic stroke (CES), large artery stroke (LAS), and small vessel stroke (SVS)) [21,22,23,24,25], as well as for several metabolic diseases, including type 2 diabetes (T2D), gout (Gout is often associated with metabolic diseases due to its underlying cause, hyperuricemia, a known metabolic disorder. By including gout in our study, which is a form of arthritis resulting from hyperuricemia, we acknowledge its metabolic associations.), urate (urate was also included in our study due to its significant association with metabolic disorders), hyperuricemia (Characterized by abnormally high levels of urate in the serum, underscores this relationship. The pivotal role of urate, especially in the onset of gout and other metabolic arthritic conditions, highlights its relevance in the context of metabolic disorders.), and obesity (BMI) from publicly available large-scale GWAS or meta-analyses [26,27,28] (Table 1).

### 2.3. Instrumental Variables

To ensure the accuracy and validity of the causal inferences, we implemented quality control measures to select instrumental variables (IVs). SNPs associated with mineral nutrients that reached the genome-wide significance threshold of *p* < 5.0 × 10^−8^ were selected as potential IVs. To explore a more comprehensive causal relationship between the gut microbiota and disease, we used a higher threshold (*p* < 5.0 × 10^−6^) to obtain more gut microbiota IVs.

Linkage disequilibrium (LD) is the genetic linkage between different loci, meaning that genetic variation at these loci is interdependent. During MR analyses, a strong LD relationship between selected IVs can result in biased or inaccurate effect estimates. We conducted a clumping process to estimate LD, with the LD threshold for clumping set at r^2^ < 0.001 and the clumping window size set at 10,000 kb.

The validity of MR analysis depends on meeting three key assumptions, one of which is the absence of genetic variants associated with potential confounding factors. However, the presence of horizontal pleiotropy may interfere with the validity of this assumption, which can lead to biased MR results. To address this issue, we utilized MR-PRESSO and MR-Egger regression tests to detect and correct bias resulting from horizontal pleiotropy. The MR-PRESSO outlier test conducts a regression analysis on each genetic variant to identify and remove outlier observations, and then recursively repeats the process using the data without those observations. The MR-PRESSO global test is repeated recursively until the *p*-value is no longer significant (*p*-value > 0.05) [29].

To reduce the bias due to weak instrumental variables, we also calculated the F-statistic, which assesses the strength of association between instrumental variables and exposure factors. There are no weak IVs when the F-statistic is > 10 [30].

### 2.4. MR Estimates

We used the Wald ratio test to estimate the association between exposure features containing only one IV and outcome [31]. For exposure features containing two or more IVs, we used five popular MR methods: inverse-variance weighted (IVW) test, MR-Egger, weighted mode, weighted median estimator (WME), and simple mode. IVW was used as the primary MR effect estimator, which weights the MR effect estimates of all IVs to obtain an overall effect estimate [32,33].

To correct for the multiple hypothesis test results, we used the false discovery rate (FDR) correction with a false discovery rate of q-value <0.1 [34,35,36]. Associations between mineral nutrients, gut microbiota, and diseases were deemed suggestive when *p*-value <0.05 but q-value ≥0.1.

### 2.5. Sensitivity Analysis

To assess the robustness of the causal associations, sensitivity analyses were conducted on the results, including Cochran’s Q statistics, MR-Egger intercept tests, and leave-one-out analyses. Cochran’s Q statistics were used for heterogeneity testing. The presence of heterogeneity can be implied when the Q statistic is significant at a *p*-value <0.05 [37,38]. The MR-Egger intercept test was used to assess horizontal pleiotropy. The MR effect estimates of IVs will present a non-zero intercept in the presence of horizontal pleiotropy. The MR-Egger intercept tests whether the non-zero intercept is significant by fitting a regression model with an intercept term and a slope term [39]. Leave-one-out analyses are used to assess the contribution of each IV to the overall effect estimate and the impact of robustness.

All statistical analyses were performed using R version 4.2.1. MR analyses were performed using TwosampleMR, MR-PRESSO, and the q-value R package.

## 3. Results

In this study, we conducted a series of quality control steps and obtained SNPs to be used as IVs for each serum mineral nutrient feature (calcium, copper, iron, magnesium, and zinc) and the gut microbiota features at the phylum, class, order, family, and genus levels. For all causal associations, the F-statistic for IV was >10, thus there was no weak IV bias. Details of the specific IVs are provided in Additional File S1: Tables S1–S5.

### 3.1. Causal Association of Mineral Nutrients and Gut Microbiota on Neurological, Psychiatric, and Metabolic Diseases

**Figure 2:**
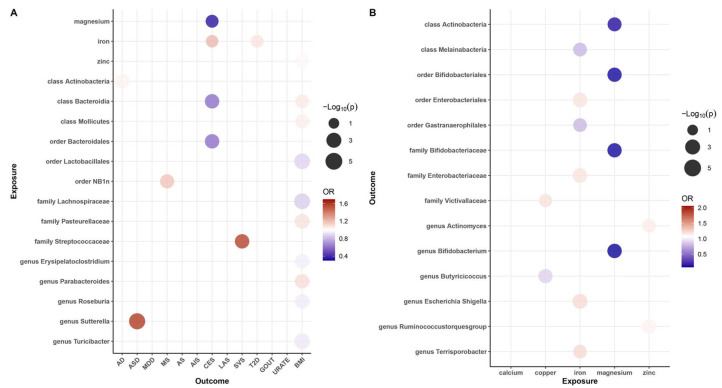
Balloon plot of the association between exposure and outcome. (**A**) Balloon plot of the association between gut microbiota and diseases (*p*-value < 0.05 and q-value < 0.10). (**B**) Balloon plot of the association between mineral nutrition and gut microbiota. Red represents a positive correlation; blue represents a negative correlation, with OR = 1 as the threshold.

**Figure 3:**
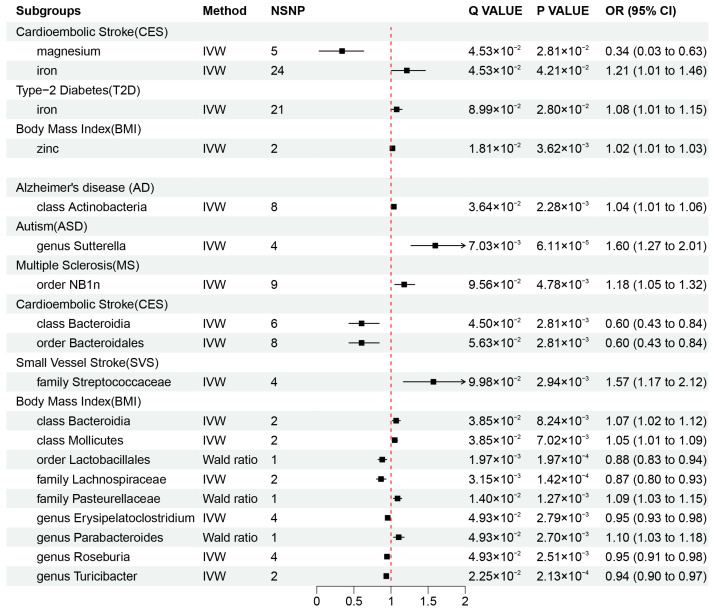
Mendelian randomization results of causal effects between mineral nutrients, gut microbiota, and diseases (*p*-value < 0.05 and q-value < 0.10). OR, odds ratio; CI, confidence interval; IVW, inverse variance weighted.

Our results revealed that zinc (OR = 1.02, 95% CI: 1.01–1.03, *p* = 3.62 × 10^−3^, q = 1.81 × 10^−2^) may be associated with an increased risk of obesity, while iron was also found to be associated with an increased risk of CES (OR = 1.21, 95% CI: 1.01–1.46, *p* = 4.21 × 10^−2^, q = 4.53 × 10^−2^) and T2D (OR = 1.08, 95% CI: 1.01–1.15, *p* = 2.80 × 10^−2^, q = 8.99 × 10^−2^), and magnesium (OR = 0.34, 95% CI: 0.03–0.63, *p* = 4.53 × 10^−2^, q = 2.81 × 10^−2^) was found to be protective factor against CES risk. These causal associations remained significant after FDR correction (Figure 2A and Figure 3). In addition, there were two suggestive associations of calcium on ASD and iron on AS, which were no longer significant after FDR correction (Additional File S1: Table S6).

Additionally, we identified a total of 15 causal associations between 14 bacterial features and 6 disease features (Figure 2A and Figure 3), as well as 93 suggestive associations between other bacterial features and diseases (Additional File S1: Table S7). The IVW estimates of the class *Actinobacteria* (OR = 1.04, 95% CI: 1.01–1.06, *p* = 2.28 × 10^−3^, q = 3.64 × 10^−2^) showed an association with an increased risk of AD, the genus *Sutterella* (OR = 1.60, 95% CI: 1.27–2.01, *p* = 6.11 × 10^−5^, q = 7.03 × 10^−3^) indicated an association with an increased risk of ASD, and the order *NB1n* (OR = 1.18, 95% CI: 1.05–1.32, *p* = 4.78 × 10^−3^, q = 9.56 × 10^−2^) showed an association with an increased risk of MS. IVW estimates suggests that the class *Bacteroidia* (OR = 0.60, 95% CI: 0.43–0.84, *p* = 2.81 × 10^−3^, q = 4.50 × 10^−2^) and the order *Bacteroidales* (OR = 0.60, 95% CI: 0.43–0.84, *p* =2.81 × 10^−3^, q = 5.63 × 10^−2^) had protective effects on CES. The IVW estimates for the family *Streptococcaceae* (OR = 1.57, 95% CI: 1.17–2.12, *p* = 9.98 × 10^−2^, q = 2.94 × 10^−3^) showed an association with an increased risk of SVS. In addition, we found that five bacterial features (the order *Lactobacillales*, the family *Lachnospiraceae*, the genus *Erysipelatoclostridium*, the genus *Roseburia*, and the genus *Turicibacter*) are protective factors against obesity, and four bacterial features (the class *Bacteroidia*, the class *Mollicutes*, the family *Pasteurellaceae*, and the genus *Parabacteroides*) are associated with the risk of obesity (Figure 3). These associations remained significant after FDR correction.

### 3.2. Causal Association of Mineral Nutrients on Gut Microbiota

**Figure 4:**
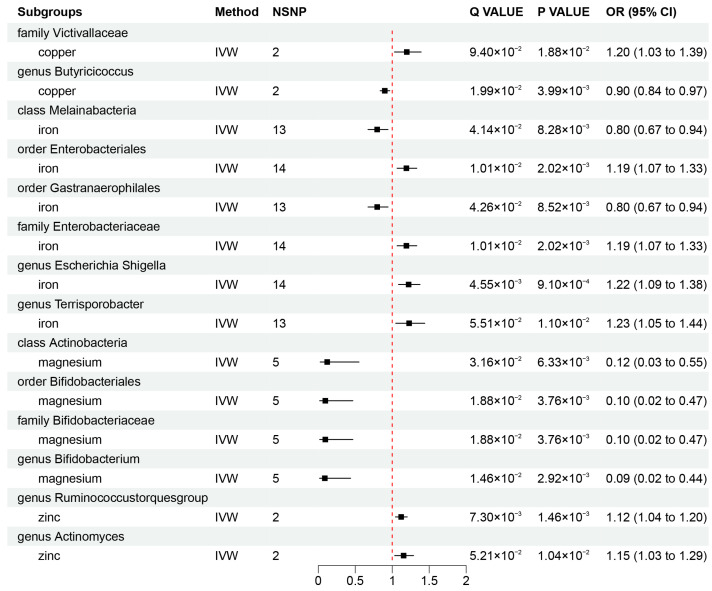
Mendelian randomization results of causal effects between mineral nutrients and gut microbiota (*p*-value < 0.05 and q-value < 0.10). OR, odds ratio; CI, confidence interval; IVW, inverse variance weighted.

To investigate whether gut microbiota regulates the association between mineral nutrients and diseases, we also estimated the association between mineral nutrients and gut microbiota (Figure 2B and Figure 4). Based on the results, we found that copper was causally associated with two bacterial traits (the family *Victivallaceae* and the genus *Butyricicoccus*), iron was causally associated with five bacterial traits (the class *Melainabacteria*, the order *Gastranaerophilales*, family *Enterobacteriaceae*, the genus *Escherichia Shigella*, and the genus *Terrisporobacter*), magnesium was negatively correlated with four bacterial traits (the class *Actinobacteria*, the order *Bifidobacteriales*, the family *Bifidobacteriaceae*, and the genus *Bifidobacterium*), and zinc was positively correlated with two bacterial traits (the genus *Ruminococcustorquesgroup* and the genus *Actinomyces*). These causal relationships were still significant after FDR correction (Figure 2B and Figure 4). In addition, 23 suggestive associations of mineral nutrients on gut microbiota were also found (Additional File S1: Table S8).

**Figure 5:**
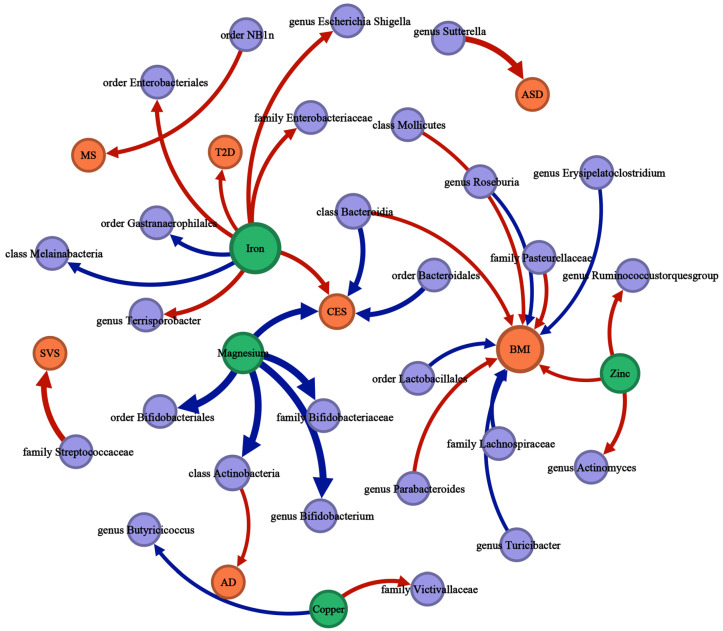
The causal associations between mineral nutrients, gut microbiota, and diseases by Mendelian randomization analysis (*p*-value < 0.05, q-value < 0.10). The thickness of the lines is positively correlated with the absolute value of the “OR-1”. The lines start with the exposure and end with the outcome. Red lines represent a positive correlation; blue lines represent a negative correlation. Green nodes represent mineral nutrient traits, purple nodes represent gut microbiota traits, orange nodes represent disease traits, and the size of the node represents the sum of the in-degree and out-degree.

Suggestive associations are not included in Figure 2, Figure 3 and Figure 4 and detailed information is available in Additional File S1: Tables S6–S8. Figure 5 is a summary network to better understand the associations between mineral nutrients, gut microbiota, and diseases.

### 3.3. Sensitivity Analyses

Through Cochran’s Q a tests, the results showed no significant heterogeneity in the IVs used for MR analysis (Additional File S1: Tables S9–S11). According to the results of the MR-Egger regression intercept analysis, all *p*-values explained by MR-Egger were >0.05, indicating that there was no significant horizontal pleiotropy (Additional File S1: Tables S12–S14). In the MR-PRESSO test, we removed outliers that had a significant level of pleiotropy and performed MR analysis for the remaining SNPs again. (Additional File S1: Tables S15–S17). The robustness of our main results was further confirmed by leave-one-out analysis (Additional File S2: Figures S1–S30).

## 4. Discussion

We conducted an MR analysis in this study to evaluate the causal associations between 5 serum mineral nutrients, 196 gut microbes, and some of neurological, psychiatric, and metabolic diseases.

Our results demonstrated the effects of iron on CES and T2D, the effect of zinc on BMI, and the effect of magnesium on CES, as well as suggestive associations of iron with AS and calcium with ASD. The associations we observed have also been reported in several previous studies.

Previous studies consistent with our results have shown that magnesium is associated with a decreased risk of CES [40,41]. Associations exist between low serum magnesium levels and elevated risks of atrial fibrillation, a strong risk factor for cardioembolic stroke. Consequently, magnesium may reduce the risk of cardioembolic stroke, attributed in part to its antiarrhythmic effects and atrial fibrillation [42]. Additionally, magnesium can also inhibit platelet aggregation and reduce the likelihood of thrombosis, and the antithrombotic effect may lead to a reduction in the risk of cardioembolic stroke [42]. Meanwhile, several clinical and animal studies have reported that magnesium has a protective role in the integrity of the blood–brain barrier and that higher serum magnesium concentration may improve the prognosis of stroke by protecting nerve cells and reducing the inflammatory response [43,44]. However, we did not observe a significant association between magnesium and other stroke subtypes, such as LAS and SVS. CES is mainly caused by thrombosis or haemorrhage, while LAS and SVS are mainly caused by lesions of the blood vessel walls [45,46,47]. Thus, magnesium might exert a more significant protective effect on CES compared to LAS and SVS. Additionally, other factors, such as lifestyle habits and disease states, may also influence the risk of different stroke subtypes, potentially modifying the effect of magnesium [48]. In contrast to magnesium, high serum iron concentration may increase the risk of CES as iron contributes to oxidative stress. Oxidative stress leads to an increased production of free radicals within cells, which results in oxidative damage to cell membranes and triggering of inflammatory responses. These reactions activate platelets and other cells, promoting platelet aggregation. Furthermore, oxidative stress can lead to impaired function of endothelial cells and exacerbate thrombosis [49,50].

In addition to the mineral nutrients, our results also highlight the class *Bacteroidia* and the order *Bacteroidales* as protective factors against CES. The protective effect of these beneficial bacteria aligns with Yin’s study [51]. *Bacteroidia* can ferment indigestible sugars to produce short-chain fatty acids (SCFAs) that enhance immunity and can improve cognitive and functional impairment in the brain after stroke via the gut–brain axis [52]. Therefore, the class *Bacteroidia* and the order *Bacteroidales* may reduce the risk of CES through the production of SCFAs.

High serum iron concentration may also contribute to insulin resistance and T2D by affecting fat metabolism and increasing fatty acid release and oxidation [53]. Iron overload can disrupt the production of reactive oxygen species in the islets, the stability of hypoxia-inducible factor 1α, and adenosine triphosphate synthesis, thereby impairing islet β-cell function and viability, which is detrimental to the prevention and treatment of T2D [54]. Thus, exploring interventions to lower serum iron concentration may be a novel strategy for preventing and treating CES and T2D. Multiple clinical and epidemiological studies have demonstrated that appropriate dietary changes can lower serum iron concentration [55], which may subsequently lower the risk of T2D and CES. Additionally, some medications, such as deferiprone [56], have been demonstrated to lower serum iron concentration. Therefore, controlling serum iron concentration may assist in controlling T2D and CES, and could offer insights for the development of future therapeutic strategies.

Our results underscore the significance of the gut microbiota, in addition to mineral nutrients, in disease development. Specifically, we identified *the* class *Actinobacteria, the* genus *Sutterella*, *the* order *NB1n*, and *the* family *Streptococcaceae* as risk factors for AD, ASD, MS, and SVS, respectively.

A study indicated a slightly higher abundance of *Actinobacteria* in the intestines of AD patients compared to healthy individuals [57]. However, Vogt NM et al. found a significantly lower abundance of *Actinobacteria* in AD patients [58]. This suggests that specific species and strains of actinomycetes may affect different AD patients differently, and our study linking actinomycetes to increased AD risk may offer new insights into this uncertain association. *Sutterella*, identified as a microbial biomarker for ASD patients in linear discriminant effect size analysis, is a principal bacterial genus implicated in the increased risk of ASD, a finding consistent with our results [59]. The study by Williams et al. also indicated a higher abundance of *Sutterella* in children with ASD [59,60].

Tan et al.’s study further supported our findings, and they found that the enrichment of the family *Streptococcaceae* in IS patients was positively correlated with the apolipoprotein B (ApoB)/ApoA1 ratio, a high-risk factor for IS patients, and negatively correlated with a preventive factor (high-density lipoprotein cholesterol (HDL-c)) in two ethnic minority (Tujia and Miao) and Han populations. Additionally, Tan et al. found that the enrichment of *Ruminococcaceae* and *Lachnospiraceae* in healthy populations was negatively associated with risk factors (systolic blood pressure, ApoB/ApoA1 ratio, fasting plasma glucose, and high-sensitive C-reactive protein) [61]. In our suggestive analysis, we also found the same association of *Ruminococcaceae* and *Lachnospiraceae* with IS subtypes CES and LAS.

Our analysis also revealed that many gut microbes are associated with BMI. Specifically, we identified the order *Lactobacillales*, the family *Lachnospiraceae*, the genus *Erysipelatoclostridium*, the genus *Roseburia*, and the genus *Turicibacter* as protective factors against obesity, while the class *Bacteroidia*, the class *Mollicutes*, the family *Pasteurellaceae*, and the genus *Parabacteroides* are risk factors for obesity.

Strains of the genus *Roseburia* have been found to be beneficial for individuals with high BMI, as they promote the excretion of indigestible polysaccharides when consumed by obese people [62,63,64].

High levels of HDL-c and superoxide dismutase (SOD) are beneficial for preventing, controlling, and treating obesity. Some studies have reported lower levels of HDL-c and lower total serum SOD activity in obese individuals compared to healthy individuals [65]. The mouse animal studies highlighted genus *Turicibacter* as a typical bacterium positively correlated with HDL-c and SOD levels [65,66]. These studies can support the association of genus *Turicibacter* on BMI in our results.

In addition to the association of short-chain fatty acids (SCFAs) with stroke, several clinical and animal studies have shown that SCFAs are effective in reducing BMI [67]. In this study, some of the gut microbiota associated with BMI were identified as SCFA-producing bacteria, including *Lactobacillales*, *Lachnospiraceae*, and *Roseburia* [67]. The beneficial metabolic effects of SCFAs are mediated by adipose peroxisome proliferator-activated receptor-γ, including preventing high-fat diet-induced (HFD)-obesity and improving insulin sensitivity [68]. Moreover, butyrate and propionate inhibit food intake, curb HFD-induced weight gain and glucose intolerance, and stimulate intestinal hormone secretion, mainly through free fatty acid receptor 3-independent mechanisms [69]. Chih min Chiu collected 81 stool samples from Taiwanese participants and found a positive correlation between *Parabacteroides distasonis* and obesity; this association is consistent with our findings [70]. Additionally, we also found a positive correlation between zinc and BMI in our results.

According to this study on the causal associations of mineral nutrients on gut microbiota, we found that magnesium is negatively correlated with the order *Bifidobacteriales*, the family *Bifidobacteriaceae*, and the genus *Bifidobacterium*. This supports previous findings that magnesium deficiency alters *Bifidobacteria* concentrations in the gut, underscoring a notable negative relationship between magnesium levels and *Bifidobacteria* [71]. Additionally, we found associations between iron and *Escherichia Shigella* and *Enterobacteria* have also been noted in other studies, where providing iron-containing micronutrient powder (MNP) to weaned infants affected the gut microbiota, with +FeMNP increasing the abundance of *Shigella* and *Enterobacteria* [72]. Furthermore, the positive association between iron and *Enterobacteriaceae* was demonstrated in a mouse model [73,74]. We also found a negative correlation between iron and *Melainabacteria* and *Gastranaerophilales*, which may be due to different gut microbes having varying requirements and metabolisms of iron. For example, many pathogenic *Enterobacteriaceae* are dependent on iron for bacterial virulence or colonisation of the gut [75,76].

Zinc, a crucial nutrient, can significantly alter the distribution and function of the microbiota. Zinc-binding proteins account for 5–6% of the bacterial proteome, highlighting the critical importance of zinc for the composition of microorganisms in the gut [77,78]. It has been reported that the long-term consumption of a zinc-rich diet significantly increases *Actinobacteria* levels in mice’s gut [77,78,79], and our study confirms the positive correlation between zinc and the *Actinomyces* genus, which is a genus within the phylum *Actinobacteria*. Our study focusing on the genus level may provide a more precise reference compared to previous studies.

A mouse model study found that dietary magnesium levels initially had a positive correlation with *Bifidobacteria* abundance, but 21 days later, this correlation turned negative [80]. Initially, increased magnesium concentration may stimulate *Bifidobacteria* growth. However, the excess of magnesium could have either negatively impacted the *bifidobacteria* or prompted the bacteria to reduce their magnesium dependence through physiological or metabolic adaptations. The results observed after 21 days are consistent with our findings. This dynamic self-regulation potentially involves complex physiological and metabolic mechanisms. To fully understand its operation, further research is required.

After integrating all significant associations (*p*-value < 0.05, q-value < 0.10), we assessed the mediating role of the gut microbiota in the effects of serum mineral nutrients on neurological, psychiatric, and metabolic diseases; however, we did not find that any of the gut bacteria were identified as mediators. Subsequently, we included suggestive associations (*p*-value < 0.05, q-value > 0.10) and found associations of “zinc-family *Pasteurellaceae*-BMI” and “Calcium-genus *Coprobacter*-ASD”, but the indirect effect (log(OR_1_) × log(OR_2_)) of zinc/calcium on BMI/ASD via family *Pasteurellaceae*/genus *Coprobacter* was not in the same direction as the direct effect (log(OR_3_)) of zinc/calcium on BMI/ASD. Therefore, we do not consider the family *Pasteurellaceae* or the genus *Coprobacter* as mediators. In conclusion, in our study, we found no evidence to support that any of the gut bacteria mediated the association between specific mineral nutrients and disease.

However, there are some limitations in this study. The gut microbiota was chosen as the exposure for MR with the limitation that the abundance of gut microbiota may be affected by medication, gender, diet, etc., and the variance explained by genetics may decrease, so we incorporated serum mineral nutrients directly related to diet into the exposure and analysed the effect of mineral nutrients on the gut microbiota and diseases by MR, leading to more comprehensive insights.

The majority of participants in the GWAS meta-analyses of mineral nutrients, gut microbiota, and diseases whose data were used for the MR analyses were of European origin, so the results of this study may not be fully applicable to populations of non-European origin. Although the different GWAS datasets were derived from different samples of European populations, it is still difficult to completely rule out the possibility of population stratification.

In addition, in order to obtain more comprehensive results, a significance threshold of *p* < 5 × 10^−6^ was used for the SNPs in the analyses as the IVs of gut microbial exposure, which was not used to reach the traditional GWAS significance threshold (5 × 10^−8^). Therefore, we corrected the results for FDR to limit the possibility of false positives.

Since the lowest level of classification in gut microbiota GWAS statistics is the genus, this limitation prevented us from further exploring the causal relationship between gut microbiota and disease at the species level. In addition, we only included serum mineral concentrations in the MR analyses, and subsequent studies could include more mineral nutrient-related biomarkers as exposures to investigate the potential role of diet.

In this study, we used linear MR to estimate the linear relationship between exposures and outcomes, where a change in the exposure variable resulted in a proportional change in the outcome variable. However, this approach may fail to account for non-linear relationships between exposure and outcome and fail to describe the shape of the association. In subsequent research, we can enhance this study by adopting non-linear MR [81]. This would involve collecting sample data at different levels, such as low, medium, and high levels of serum mineral concentration and calculating segmented linear MR estimates at each level. Specifically, we can determine the local average causal effect at each level to more accurately capture the non-linear relationship between exposure and outcome.

## 5. Conclusions

In summary, our results provide a comprehensive estimate of the causal effects of 5 serum mineral nutrients and 196 gut microbes on various neurological, psychiatric, and metabolic diseases, including AD, ASD, MDD, MS, AS, AIS, CES, LAS, SVS, T2D, gout, urate, and obesity. While multiple causal effects were identified, none of the gut microbes were found to mediate the effects of mineral nutrients on the evolution of the disease. This study contributes towards addressing the long-standing question of whether gut microbes mediate the association between mineral nutrients and a series of neurological, psychiatric, and metabolic diseases, and has potential implications for nutritional therapy.

## Figures and Tables

**Table 1:** Characteristics of included genome-wide association studies for diseases.

Traits	Populations	Sample Size	Reference	Number of SNPs	Year
Diseases					
AD	European	71,880 cases, 383,378 controls	Jansen, I.E. et al. [21]	13,367,299	2019
ASD	European	18,381 cases 27,969 controls	Grove, J. et al. [22]	9,112,386	2019
MDD	European East Asian	15,771 cases 178,777 controls	Giannakopoulou, O. et al. [23]	7,922,500	2021
MS	European	47,429 cases 68,374 controls	IMSGC [24]	6,276,314	2019
STROKE (AS, AIS, CES, LAS. SVS)	European	40,585 cases 406,111 controls	Malik, R. et al. [25]	8,255,860 8,451,005 8,451,005 8,306,090 8,765,828	2018
T2D	European	180,834 cases 1,159,055 controls	Mahajan, A. et al. [26]	10,454,875	2022
GOUT	European	2,115 cases 67,259 controls	Köttgen, A. et al. [27]	2,534,835	2013
URATE	European	110,347 individuals	Köttgen, A. et al. [27]	2,447,616	2013
BMI	European	681,275 individuals	Yengo, L. et al. [28]	2,336,269	2018

## Data Availability

The datasets analysed in this study can be downloaded from the websites: https://www.decode.com/summarydata/ (accessed on 1 June 2023), https://mibiogen.gcc.rug.nl/ (accessed on 1 June 2023), https://pgc.unc.edu/for-researchers/download-results/ (accessed on 1 June 2023), https://imsgc.net/ (accessed on 1 June 2023), https://megastroke.org/ (accessed on 1 June 2023), https://gwas.mrcieu.ac.uk/ (accessed on 1 June 2023), https://portals.broadinstitute.org/collaboration/giant/index.php/GIANT_consortium (accessed on 1 June 2023).

## Supplementary Materials

The following supporting information can be downloaded at: https://www.mdpi.com/article/10.3390/nu16020244/s1, Additional File S1: Supplementary Tables S1–S17; Additional File S2: Supplementary Figures S1–S30.

## Abbreviations

AD: Alzheimer’s disease; AIS: Any ischemic stroke; AS: Any stroke; ASD: Autism spectrum disorder; CES: Cardioembolic stroke; FDR: False discovery rate; GWAS: genome-wide association studies; HDL-c: high-density lipoprotein cholesterol; IV: instrumental variable; IVW: inverse-variance weighted; LAS: Large artery stroke; LD: linkage disequilibrium; MDD: Major depressive disorder; MR: Mendelian randomization; MS: Multiple sclerosis; RCTs: randomized controlled trials; SCFAs: short-chain fatty acids; SNP: Single nucleotide polymorphism; SOD: Superoxide dismutase; SVS: Small vessel stroke; T2D: Type 2 diabetes.
