# Supplementary material for: Associations between Serum Mineral Nutrients, Gut Microbiota, and Risk of Neurological, Psychiatric, and Metabolic Diseases: A Comprehensive Mendelian Randomization Study
Source: Nutrients. 2024 Jan 12;16(2):244. doi: 10.3390/nu16020244 (PMC10818407; doi:10.3390/nu16020244)

Additional File S2

Figure S1. Genetic association of iron with CES (Scatter plot and Leave-one-out plot) **2**

Figure S2. Genetic association of magnesium with CES (Scatter plot and Leave-one-out plot) **2**

Figure S3. Genetic association of iron with T2D (Scatter plot and Leave-one-out plot) **2**

Figure S4. Genetic association of zinc with BMI (Scatter plot and Leave-one-out plot) **3**

Figure S5. Genetic association of class *Actinobacteria* with AD (Scatter plot and Leave-one-out plot) **3**

Figure S6. Genetic association of genus *Sutterella* with ASD (Scatter plot and Leave-one-out plot) **3**

Figure S7. Genetic association of order *NB1n* with MS (Scatter plot and Leave-one-out plot) **4**

Figure S8. Genetic association of class *Bacteroidia* with CES (Scatter plot and Leave-one-out plot) **4**

Figure S9. Genetic association of class *Mollicutes* with BMI (Scatter plot and Leave-one-out plot) **4**

Figure S10. Genetic association of order *Bacteroidales* with CES (Scatter plot and Leave-one-out plot) **5**

Figure S11. Genetic association of family *Streptococcaceae* with SVS (Scatter plot and Leave-one-out plot) **5**

Figure S12. Genetic association of class *Bacteroidia* with BMI (Scatter plot and Leave-one-out plot) **5**

Figure S13. Genetic association of family *Lachnospiraceae* with BMI (Scatter plot and Leave-one-out plot) **6**

Figure S14. Genetic association of genus *Erysipelatoclostridium* with BMI (Scatter plot and Leave-one-out plot) **6**

Figure S15. Genetic association of genus *Roseburia* with BMI (Scatter plot and Leave-one-out plot) **6**

Figure S16. Genetic association of genus *Turicibacter* with BMI (Scatter plot and Leave-one-out plot) **7**

Figure S17. Genetic association of copper with family *Victivallaceae* (Scatter plot and Leave-one-out plot) **7**

Figure S18. Genetic association of copper with genus *Butyricicoccus* (Scatter plot and Leave-one-out plot) **7**

Figure S19. Genetic association of iron with class *Melainabacteria* (Scatter plot and Leave-one-out plot) **8**

Figure S20. Genetic association of iron with order *Enterobacteriales* (Scatter plot and Leave-one-out plot) **8**

Figure S21. Genetic association of iron with order *Gastranaerophilales* (Scatter plot and Leave-one-out plot) **8**

Figure S22. Genetic association of iron with family *Enterobacteriaceae* (Scatter plot and Leave-one-out plot) **9**

Figure S23. Genetic association of iron with genus *Escherichia Shigella* (Scatter plot and Leave-one-out plot) **9**

Figure S24. Genetic association of iron with genus *Terrisporobacter* (Scatter plot and Leave-one-out plot) **9**

Figure S25. Genetic association of magnesium with class *Actinobacteria* (Scatter plot and Leave-one-out plot) **10**

Figure S26. Genetic association of magnesium with order *Bifidobacteriales* (Scatter plot and Leave-one-out plot) **10**

Figure S27. Genetic association of magnesium with family *Bifidobacteriaceae* (Scatter plot and Leave-one-out plot) **10**

Figure S28. Genetic association of magnesium with genus *Bifidobacterium* (Scatter plot and Leave-one-out plot) **11**

Figure S29. Genetic association of zinc with genus *Ruminococcustorquesgroup* (Scatter plot and Leave-one-out plot) **11**

Figure S30. Genetic association of zinc with genus *Actinomyces* (Scatter plot and Leave-one-out plot) **11**

Figure S1. Genetic association of iron with CES (Scatter plot and Leave-one-out plot)

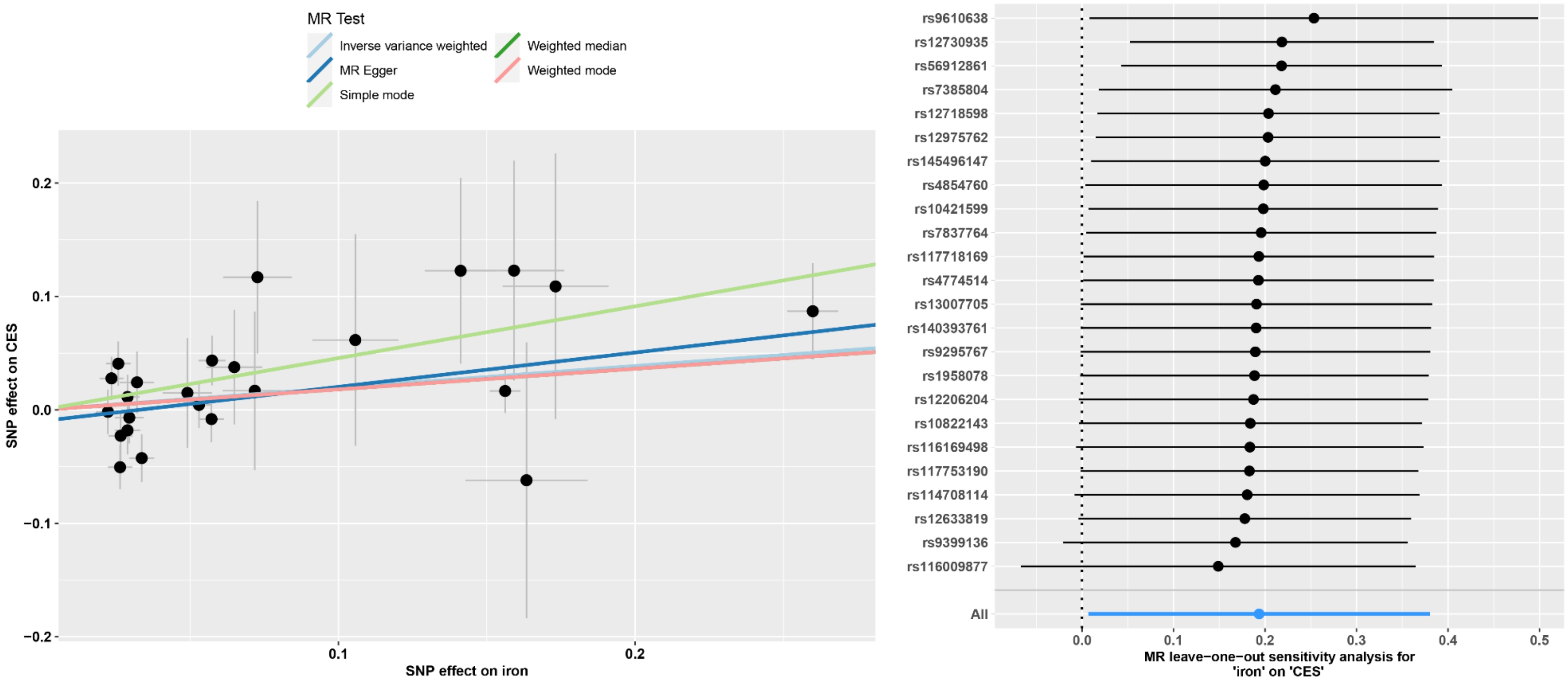

Figure S2. Genetic association of magnesium with CES (Scatter plot and Leave-one-out plot)

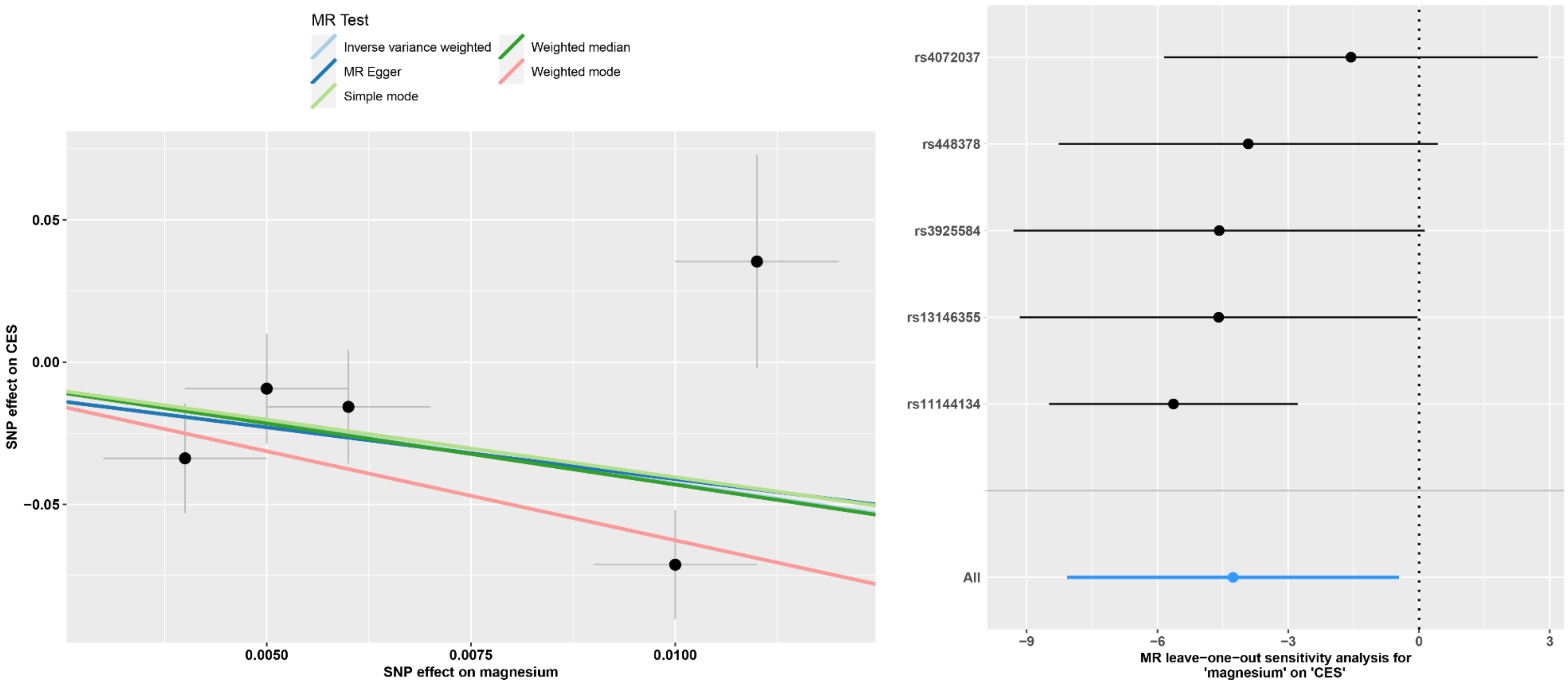

Figure S3. Genetic association of iron with T2D (Scatter plot and Leave-one-out plot)

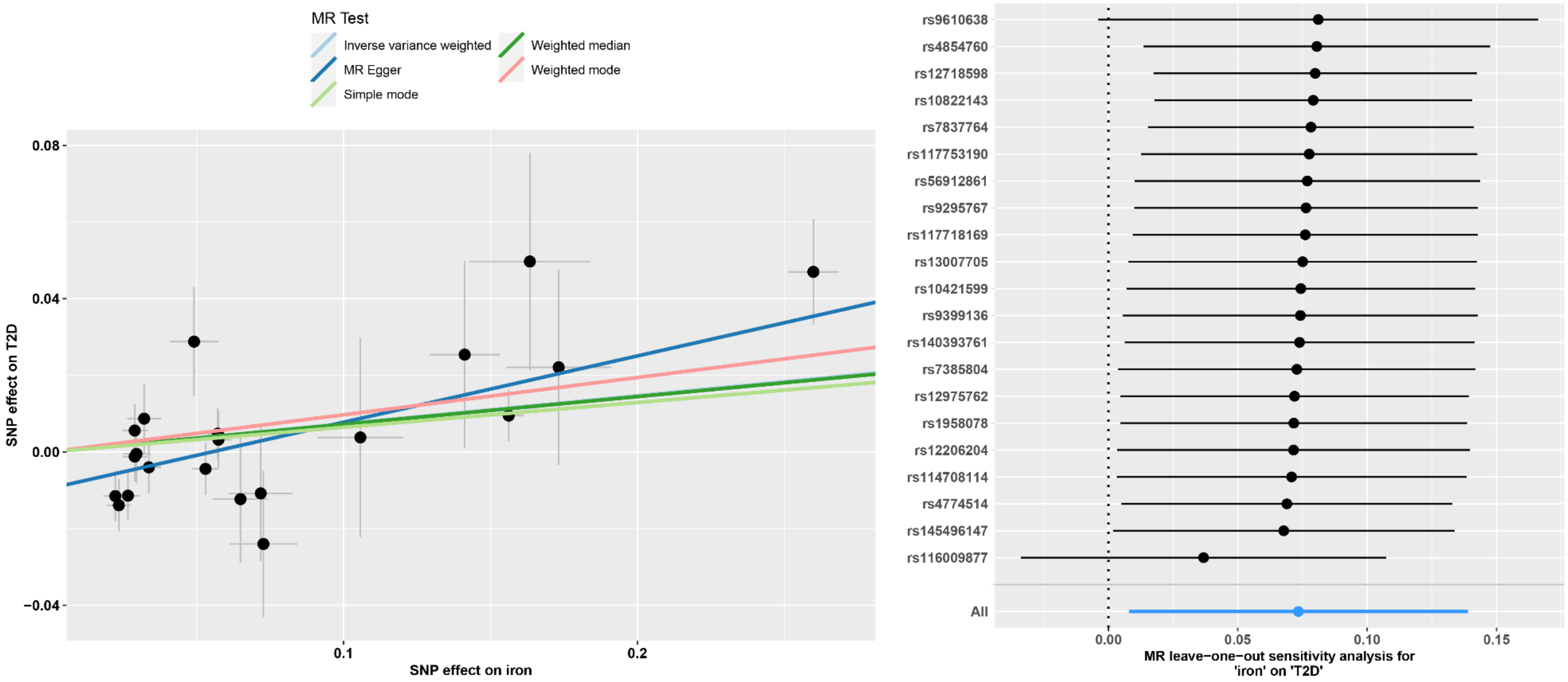

Figure S4. Genetic association of zinc with BMI (Scatter plot and Leave-one-out plot)

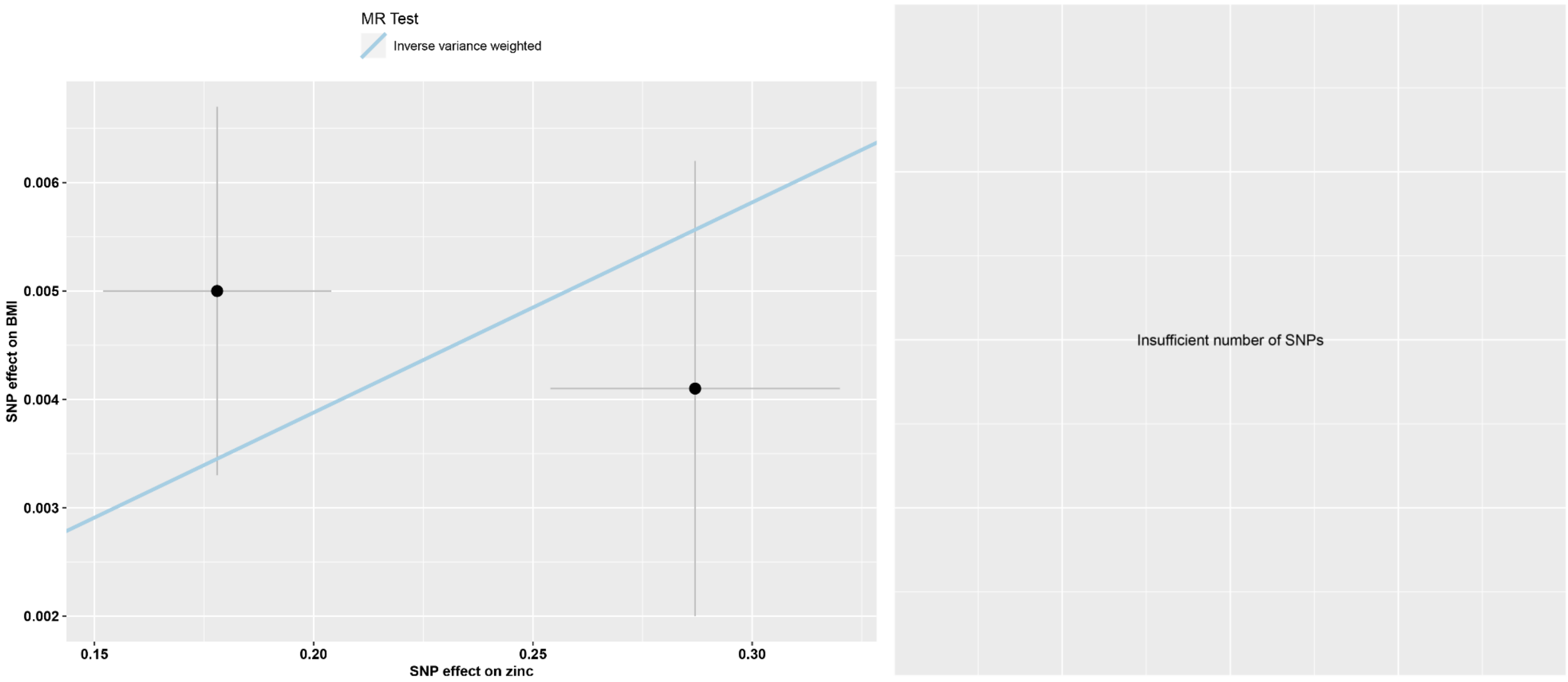

Figure S5. Genetic association of class *Actinobacteria* with AD (Scatter plot and Leave-one-out plot)

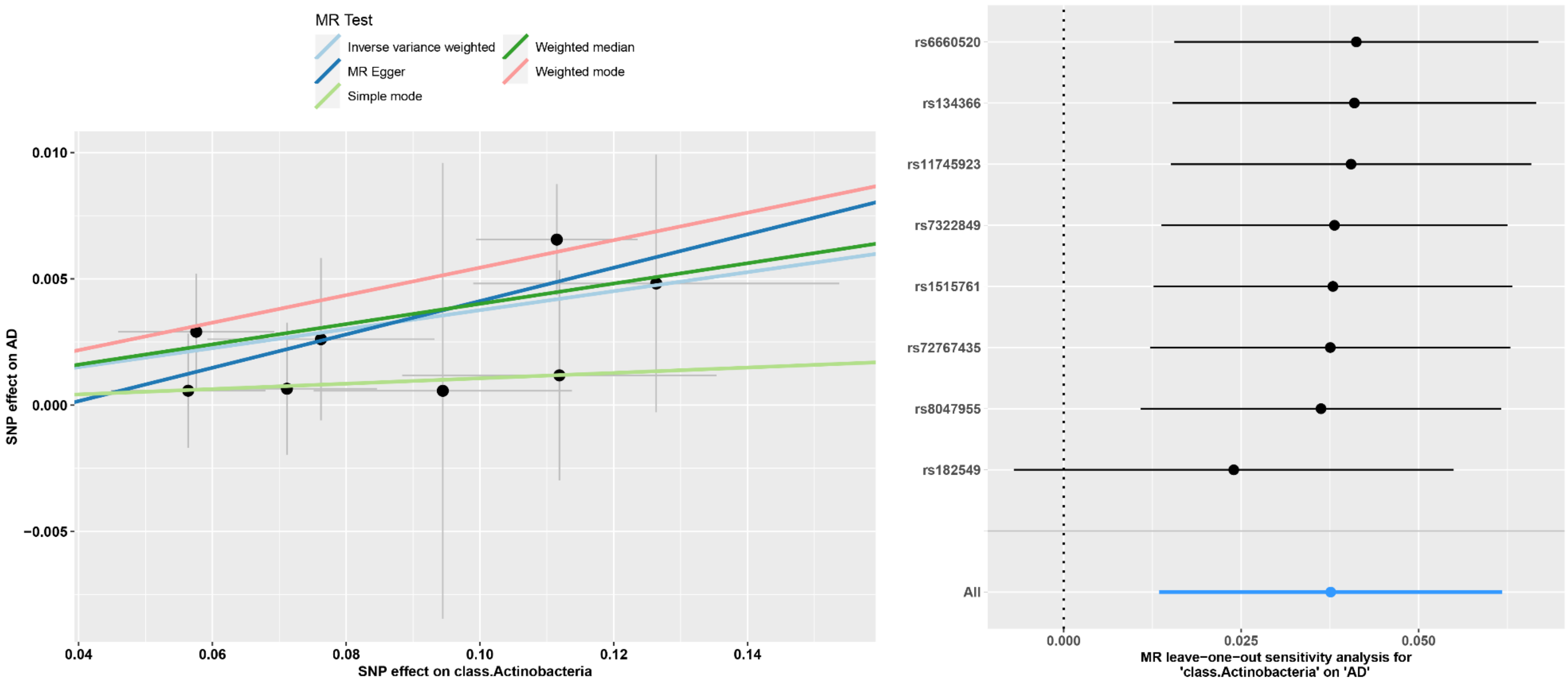

Figure S6. Genetic association of genus *Sutterella* with ASD (Scatter plot and Leave-one-out plot)

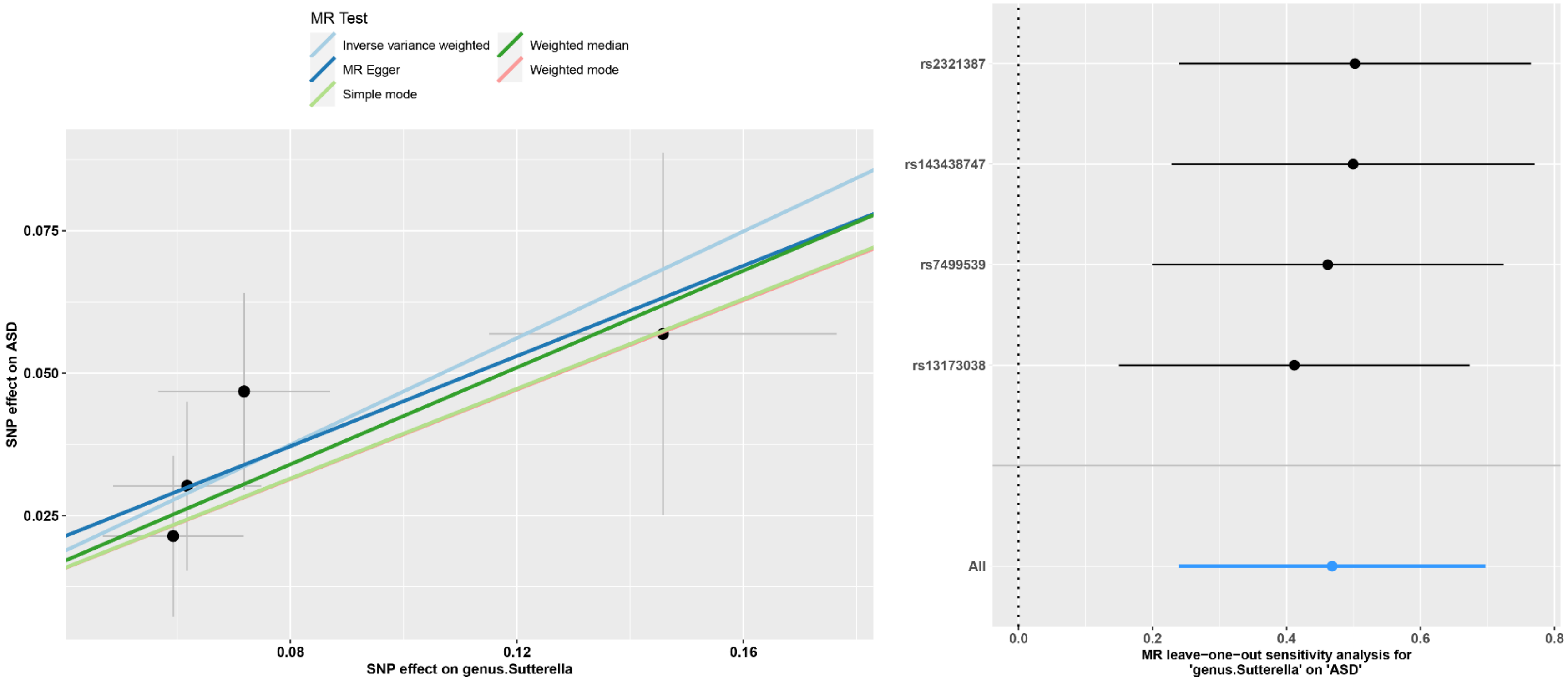

Figure S7. Genetic association of order *NB1n* with MS (Scatter plot and Leave-one-out plot)

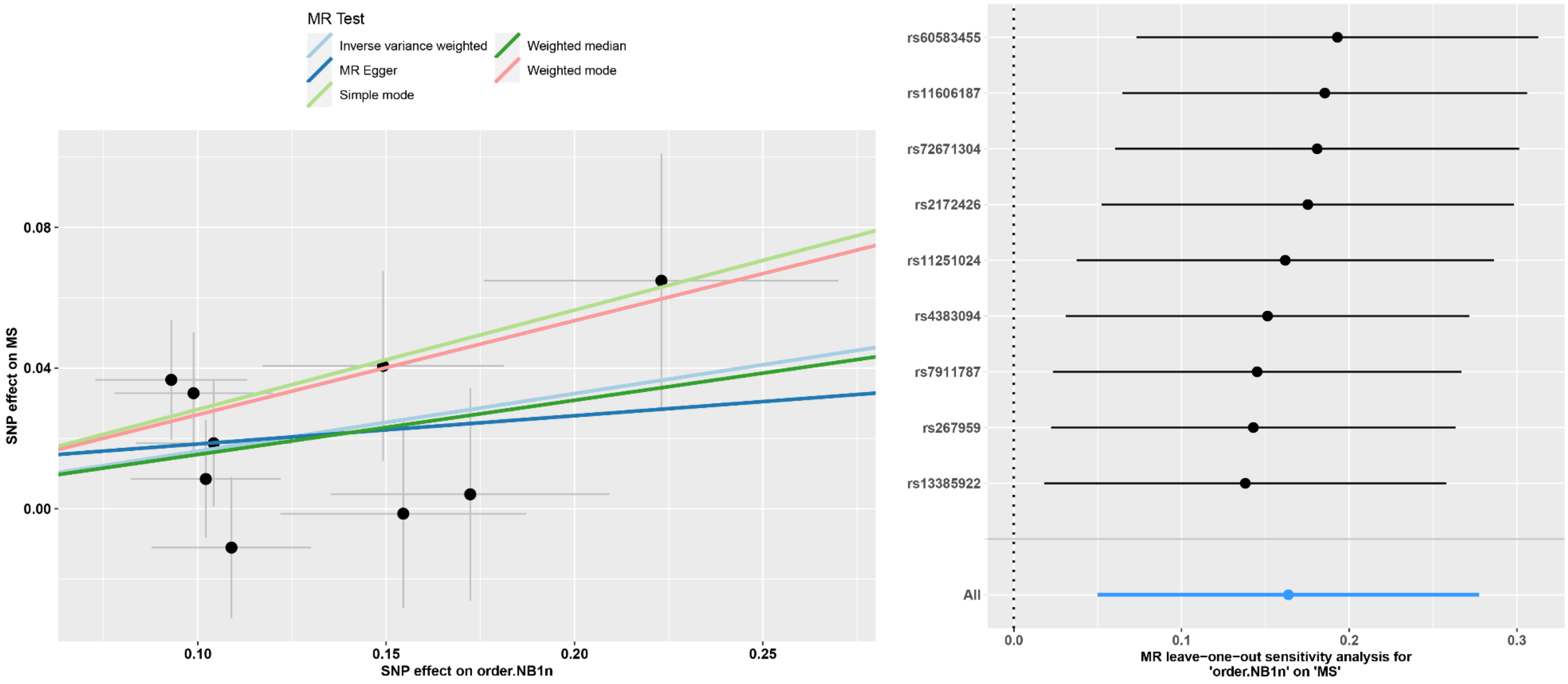

Figure S8. Genetic association of class *Bacteroidia* with CES (Scatter plot and Leave-one-out plot)

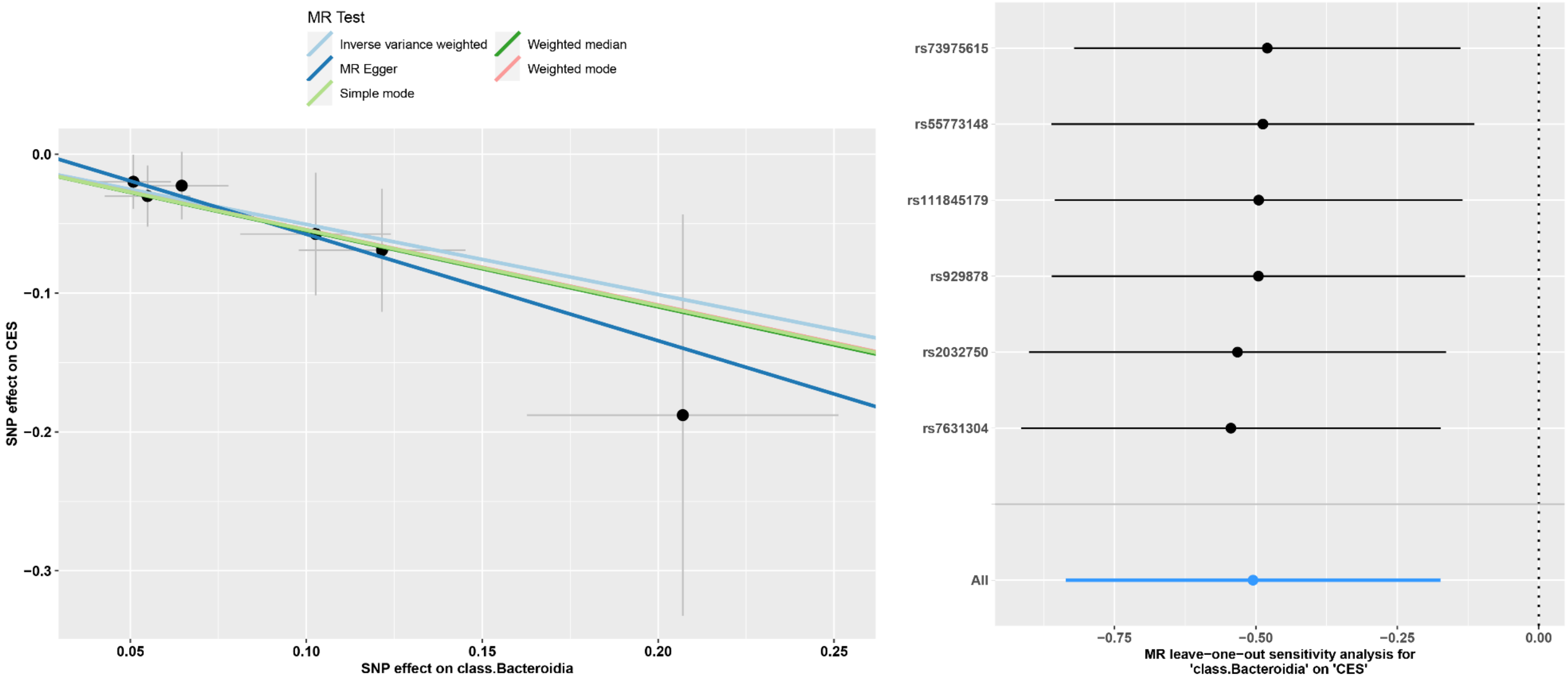

Figure S9. Genetic association of class *Mollicutes* with BMI (Scatter plot and Leave-one-out plot)

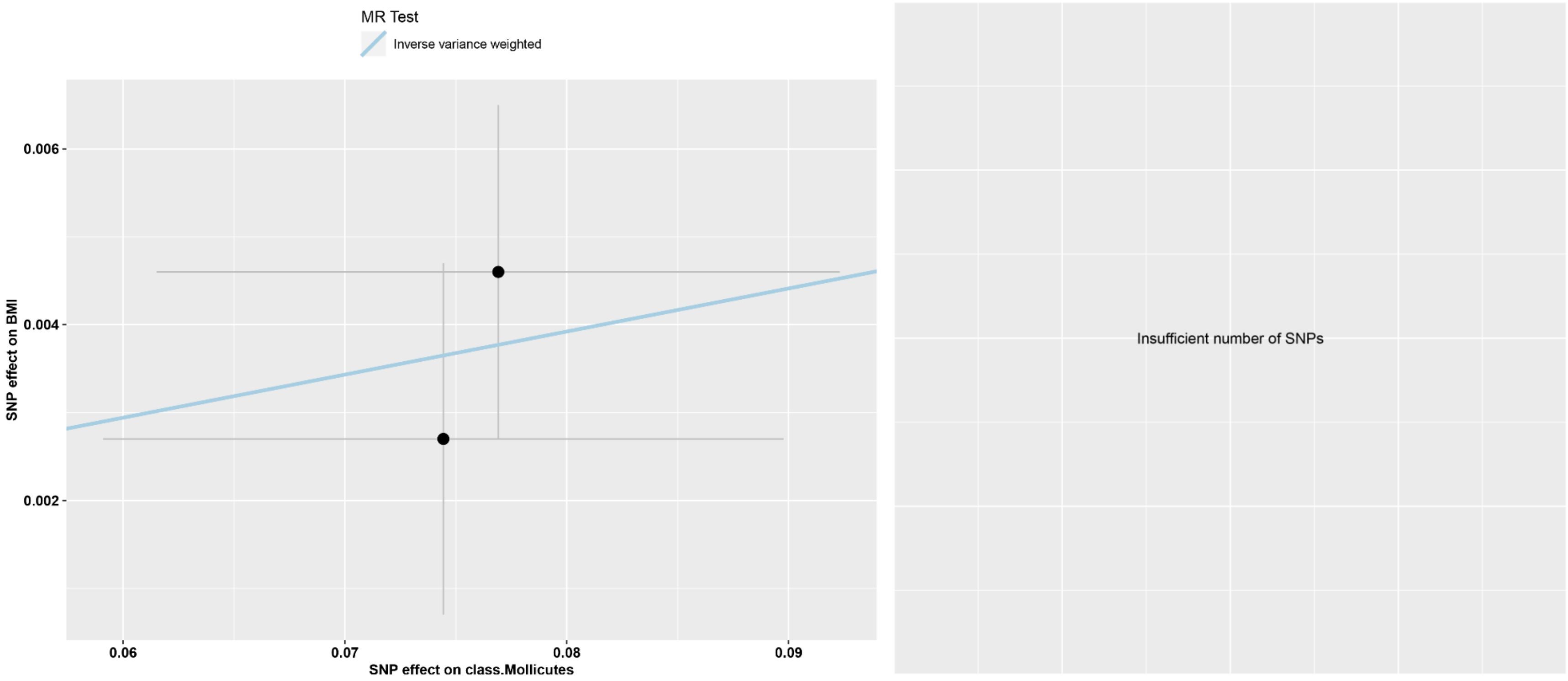

Figure S10. Genetic association of order *Bacteroidales* with CES (Scatter plot and Leave-one-out plot)

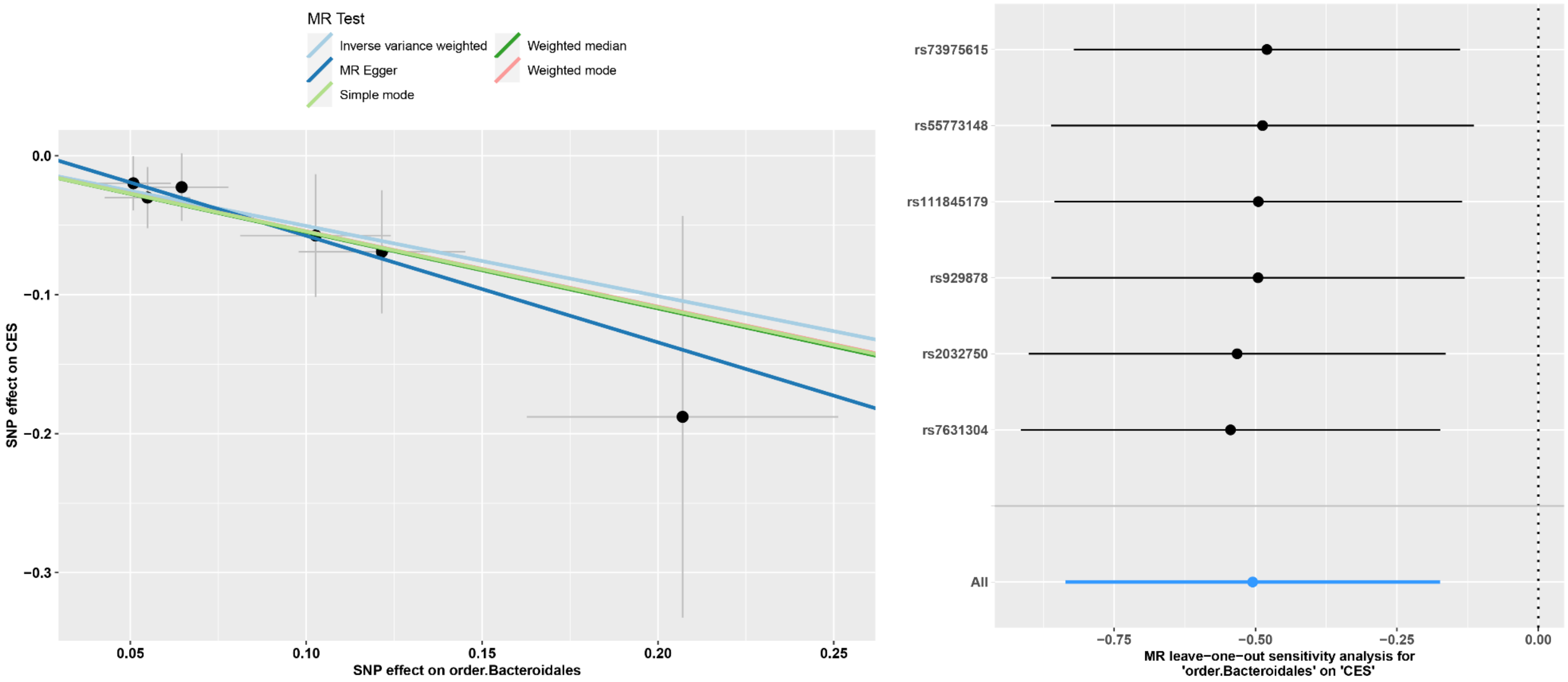

Figure S11. Genetic association of family *Streptococcaceae* with SVS (Scatter plot and Leave-one-out plot)

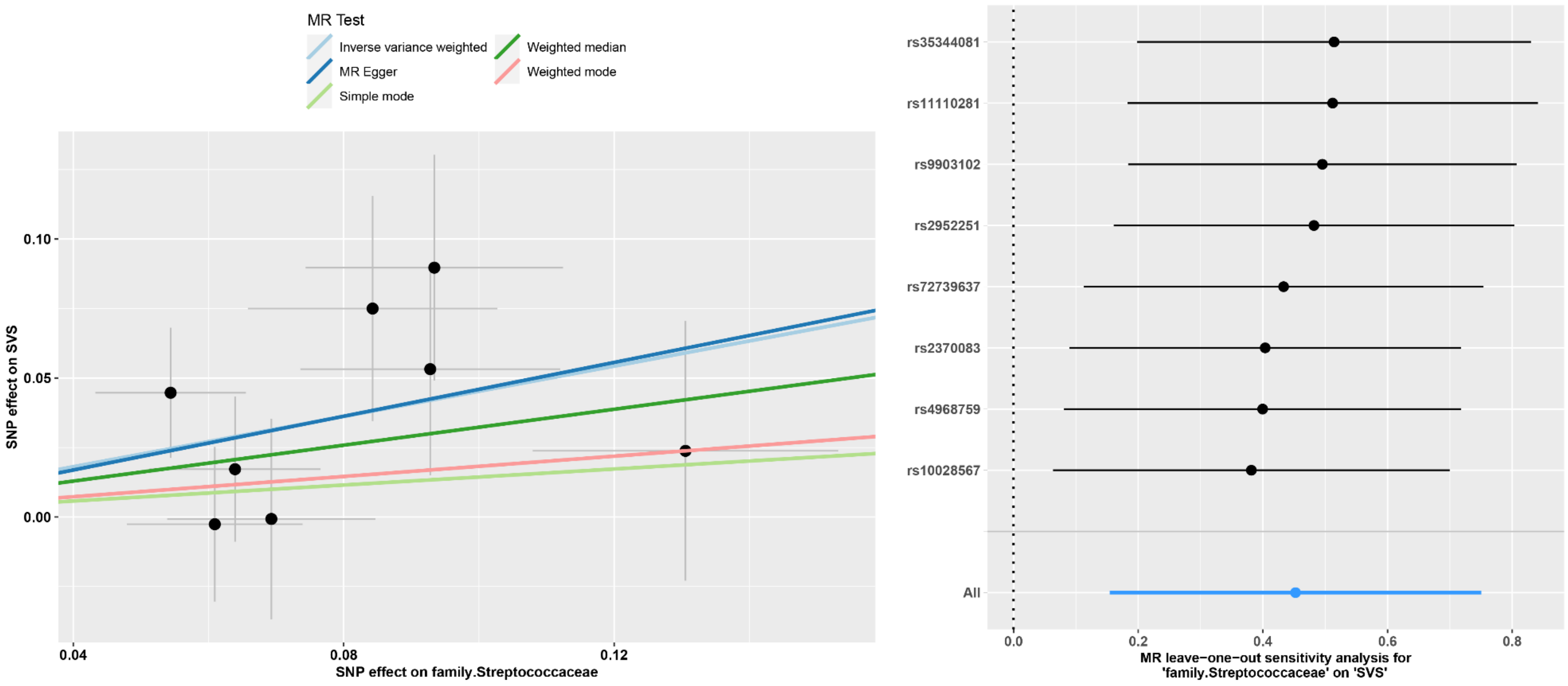

Figure S12. Genetic association of class *Bacteroidia* with BMI (Scatter plot and Leave-one-out plot)

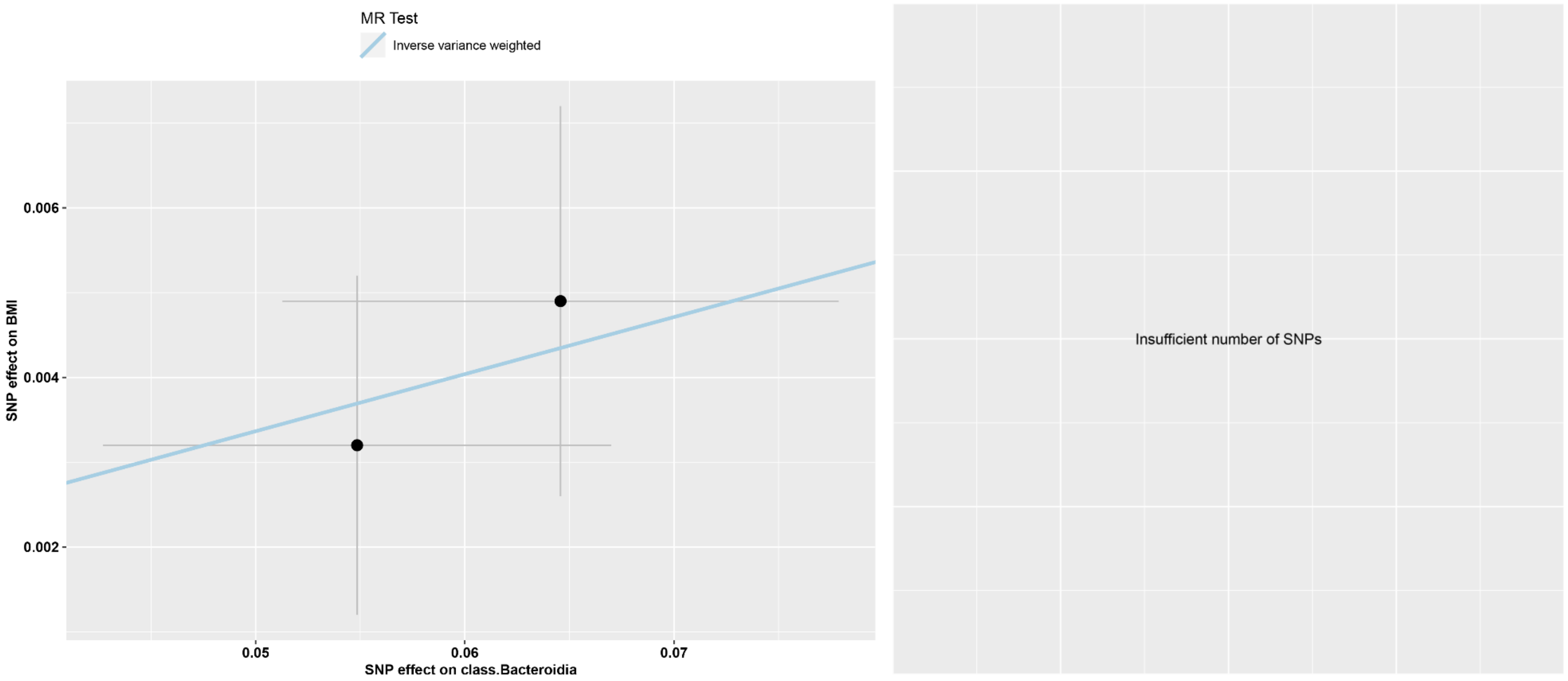

Figure S13. Genetic association of family *Lachnospiraceae* with BMI (Scatter plot and Leave-one-out plot)

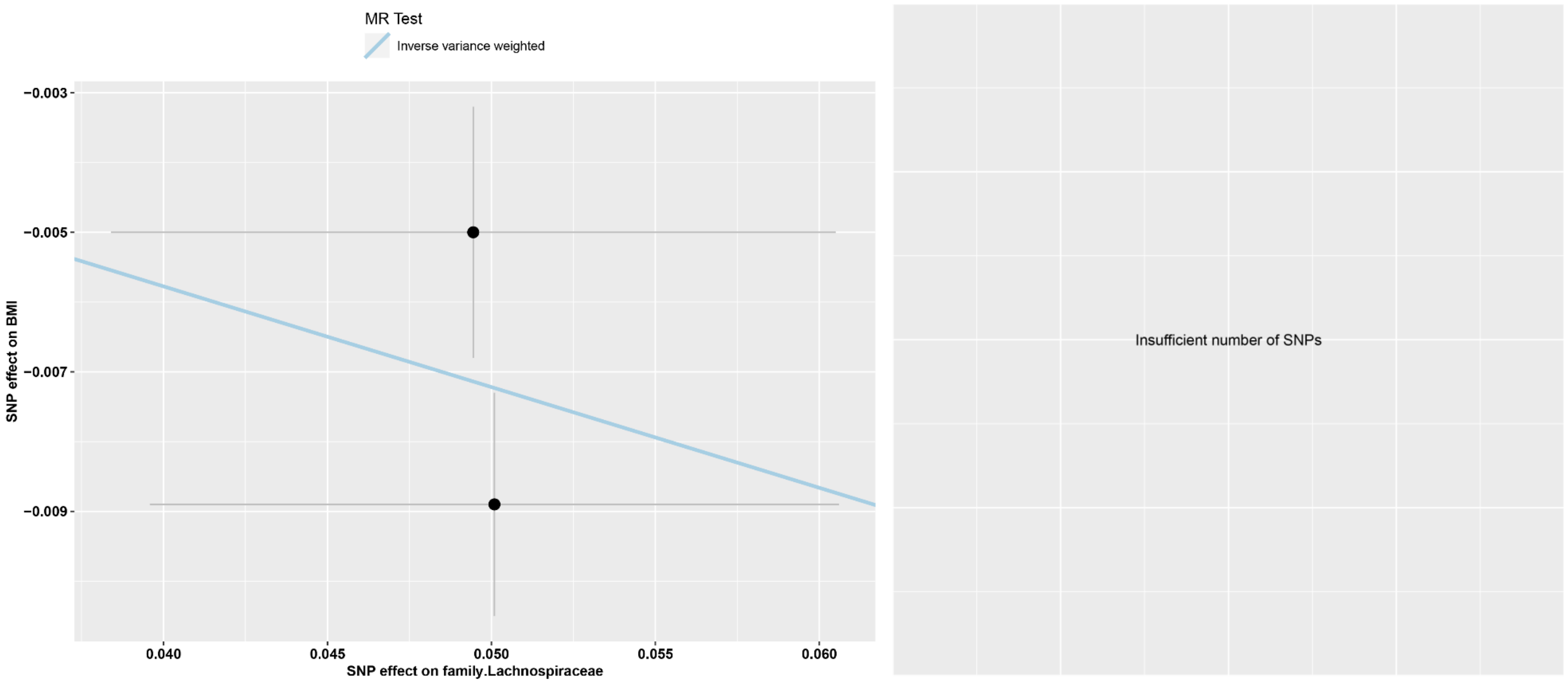

Figure S14. Genetic association of genus *Erysipelatoclostridium* with BMI (Scatter plot and Leave-one-out plot)

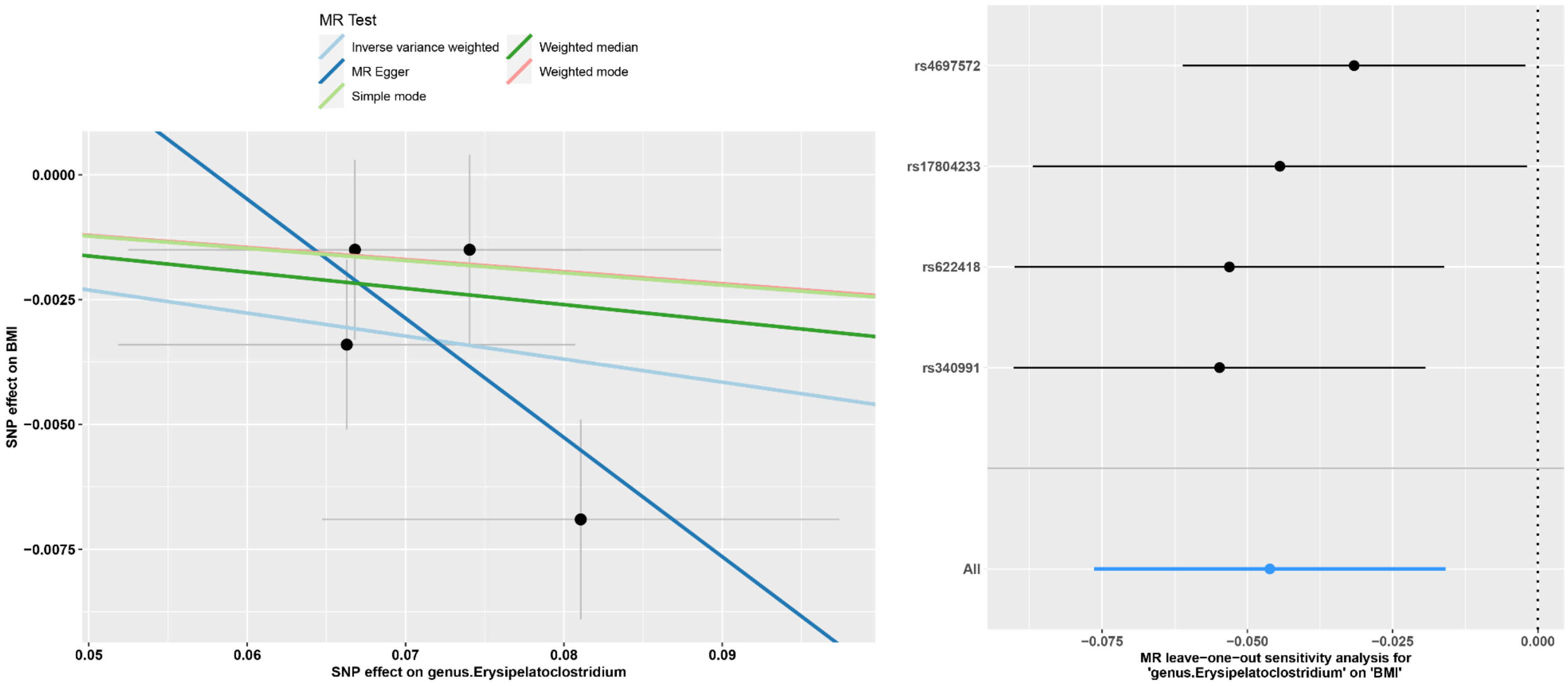

Figure S15. Genetic association of genus *Roseburia* with BMI (Scatter plot and Leave-one-out plot)

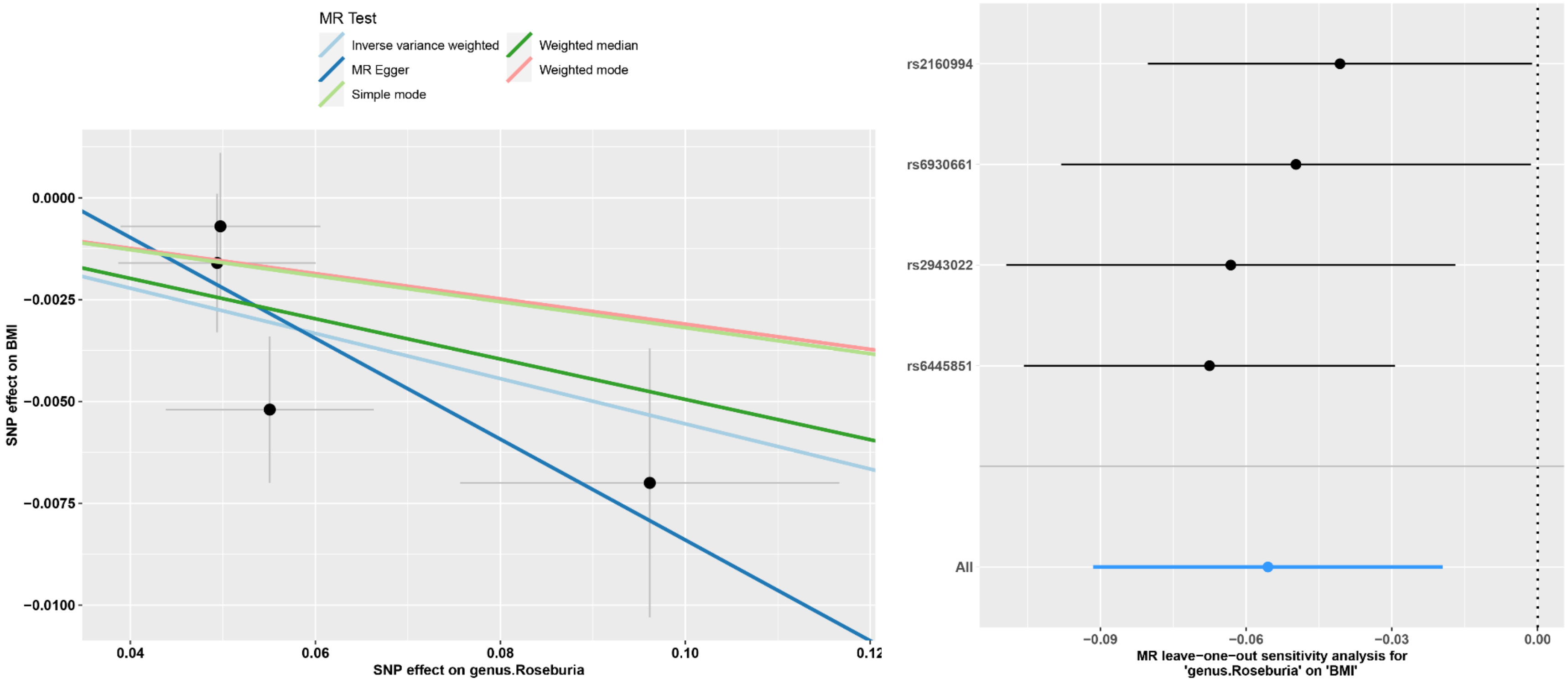

Figure S16. Genetic association of genus *Turicibacter* with BMI (Scatter plot and Leave-one-out plot)

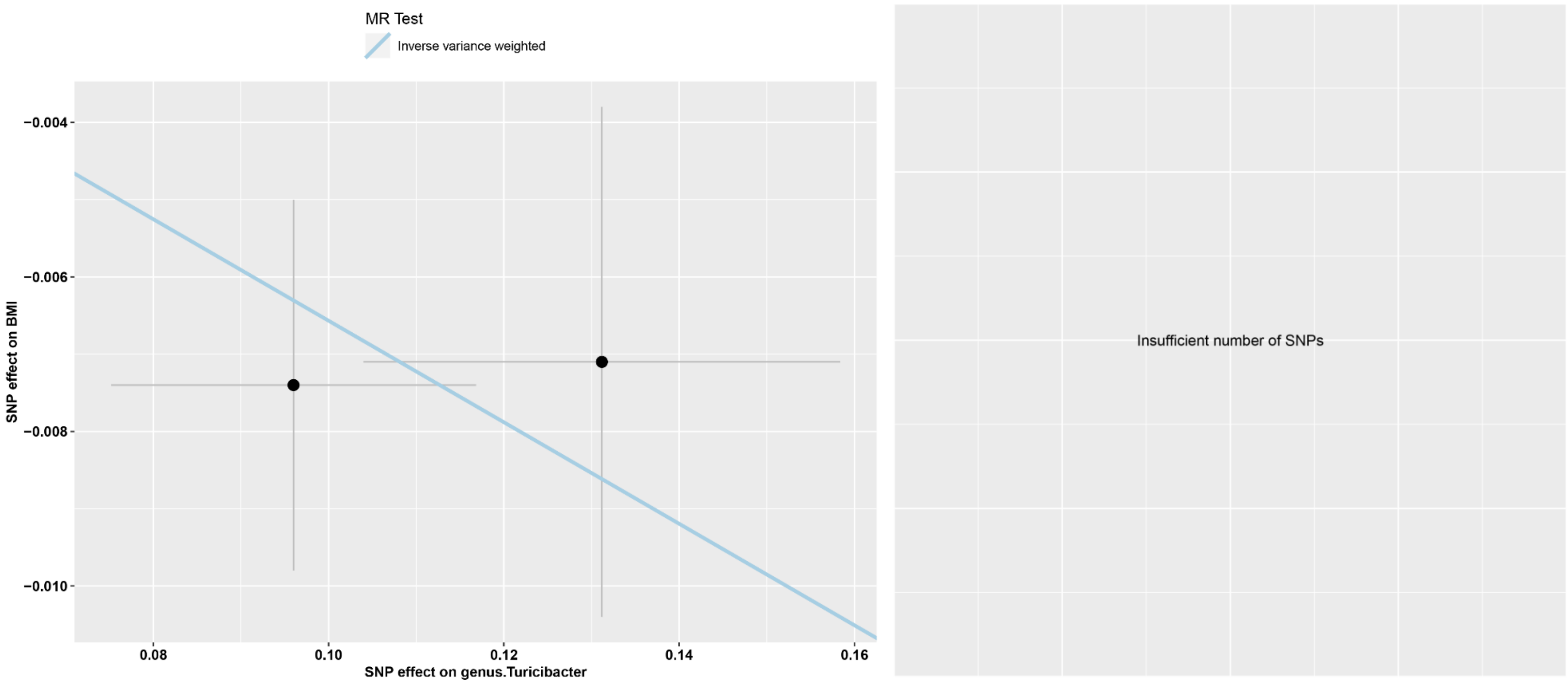

Figure S17. Genetic association of copper with family *Victivallaceae* (Scatter plot and Leave-one-out plot)

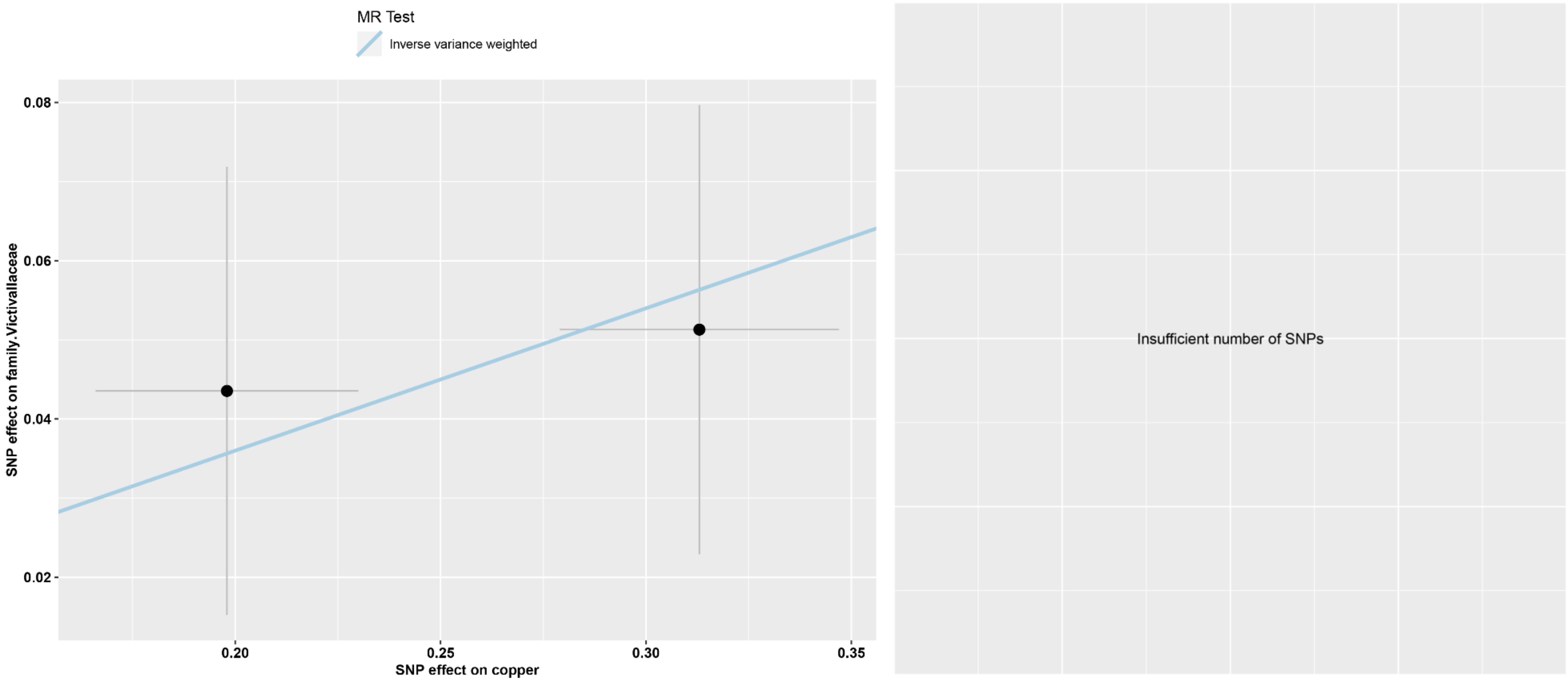

Figure S18. Genetic association of copper with genus *Butyricicoccus* (Scatter plot and Leave-one-out plot)

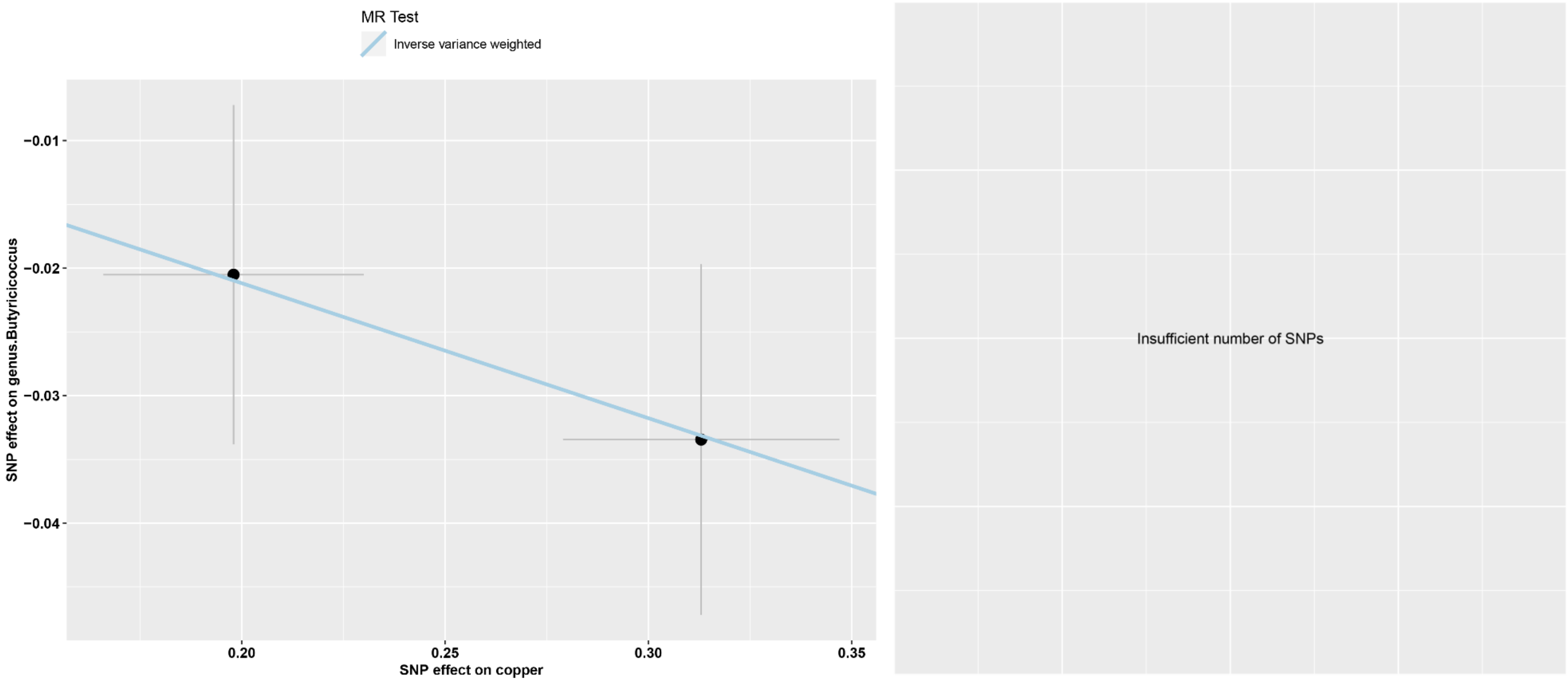

Figure S19. Genetic association of iron with class *Melainabacteria* (Scatter plot and Leave-one-out plot)

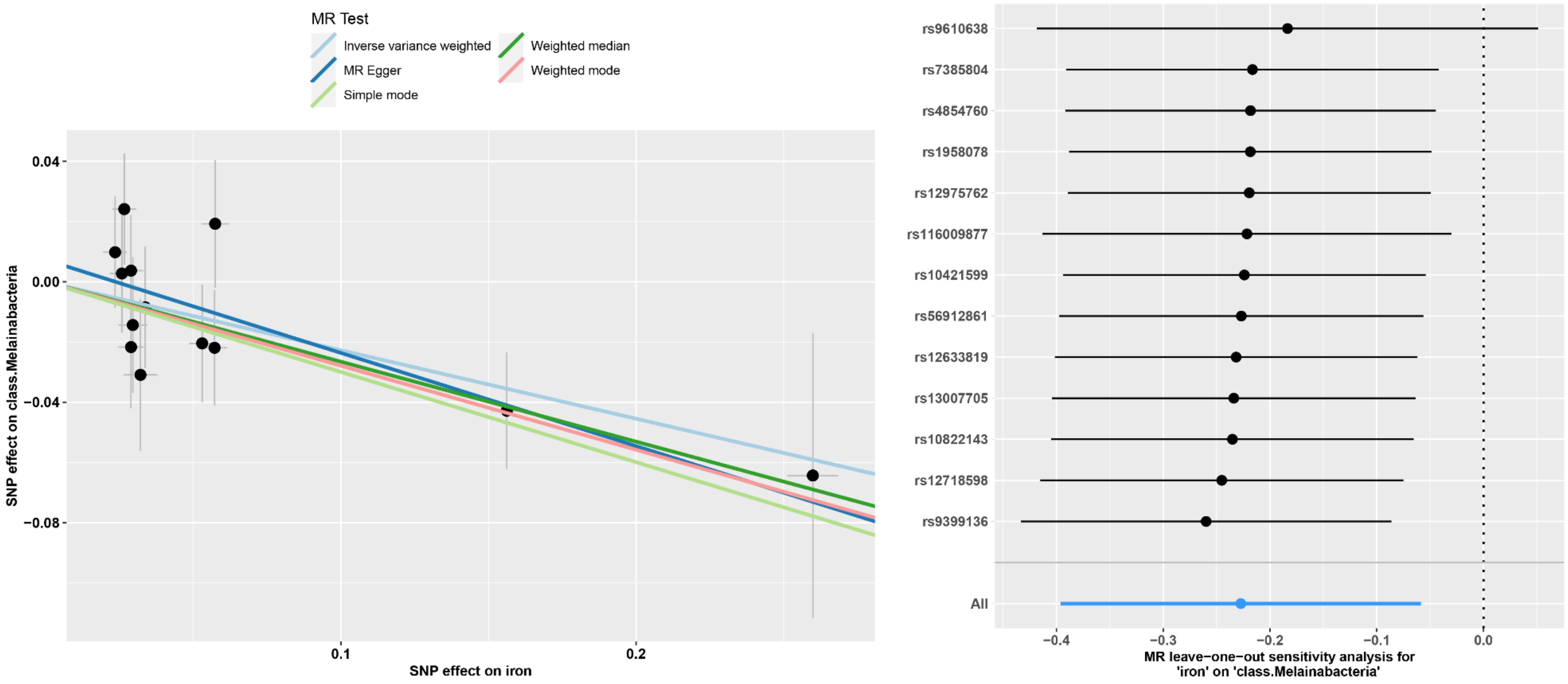

Figure S20. Genetic association of iron with order *Enterobacteriales* (Scatter plot and Leave-one-out plot)

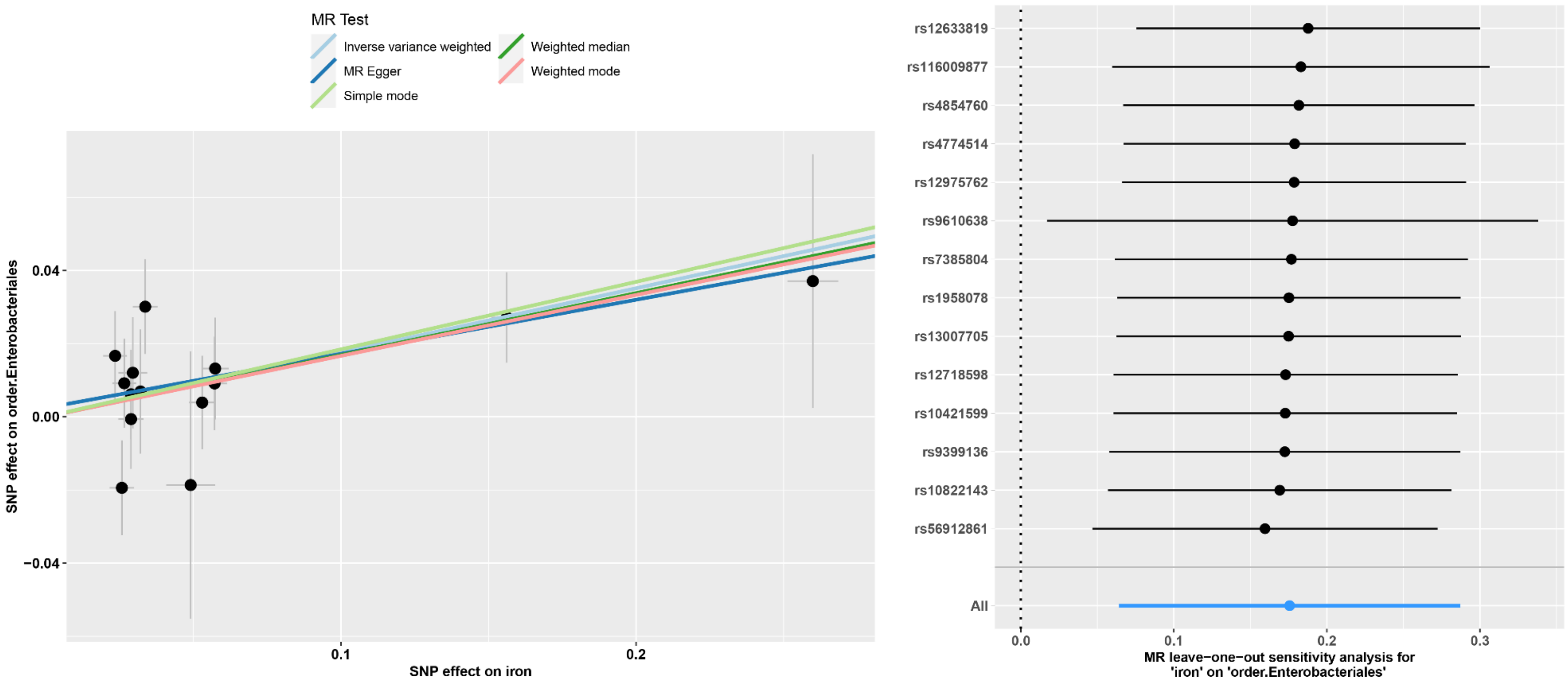

Figure S21. Genetic association of iron with order *Gastranaerophilales* (Scatter plot and Leave-one-out plot)

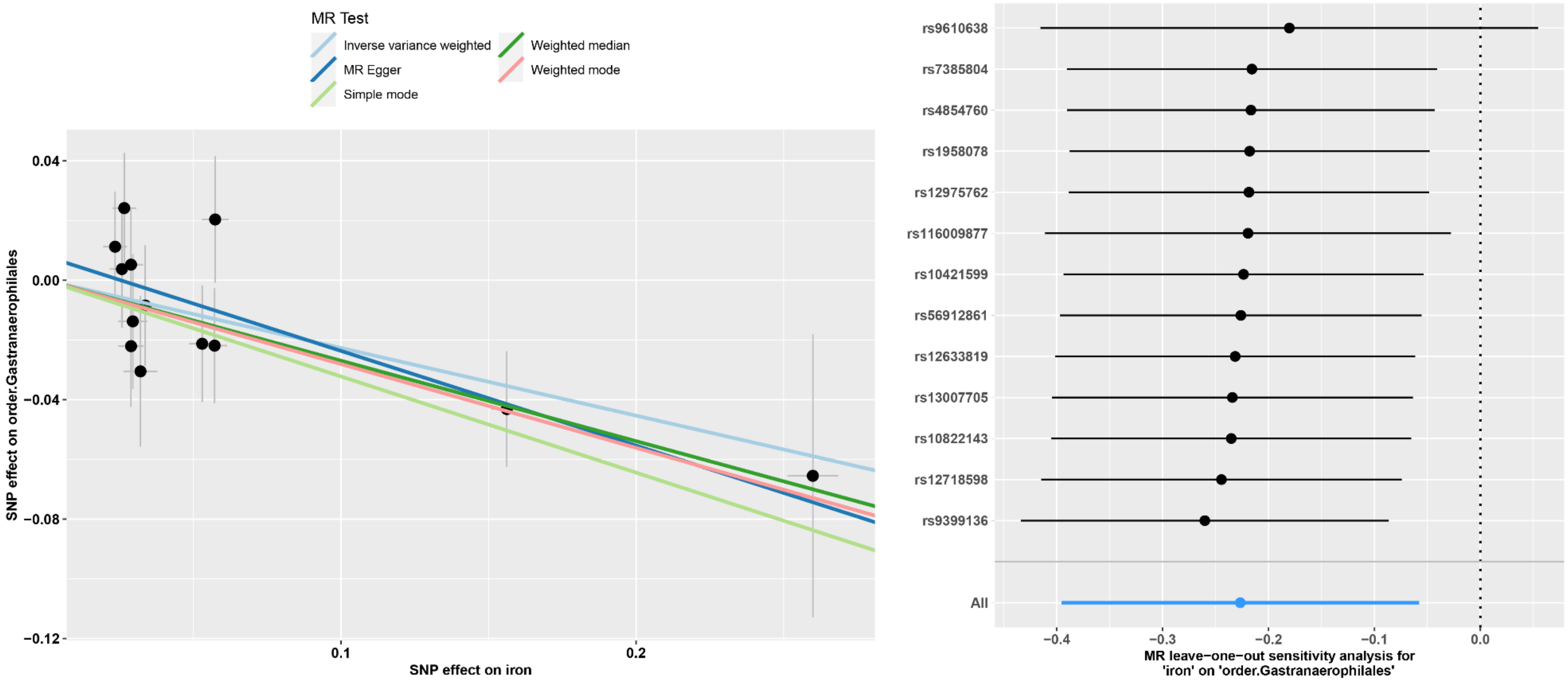

Figure S22. Genetic association of iron with family *Enterobacteriaceae* (Scatter plot and Leave-one-out plot)

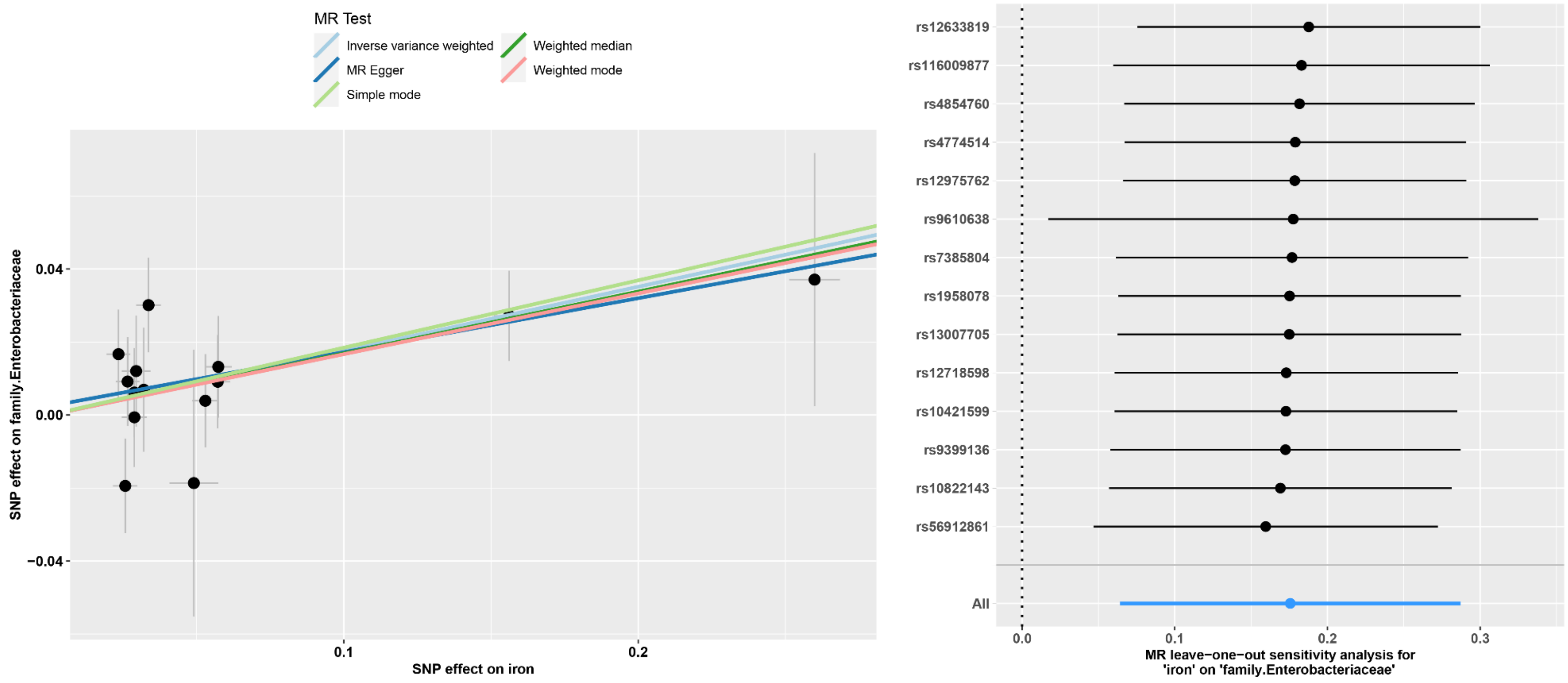

Figure S23. Genetic association of iron with genus *Escherichia Shigella* (Scatter plot and Leave-one-out plot)

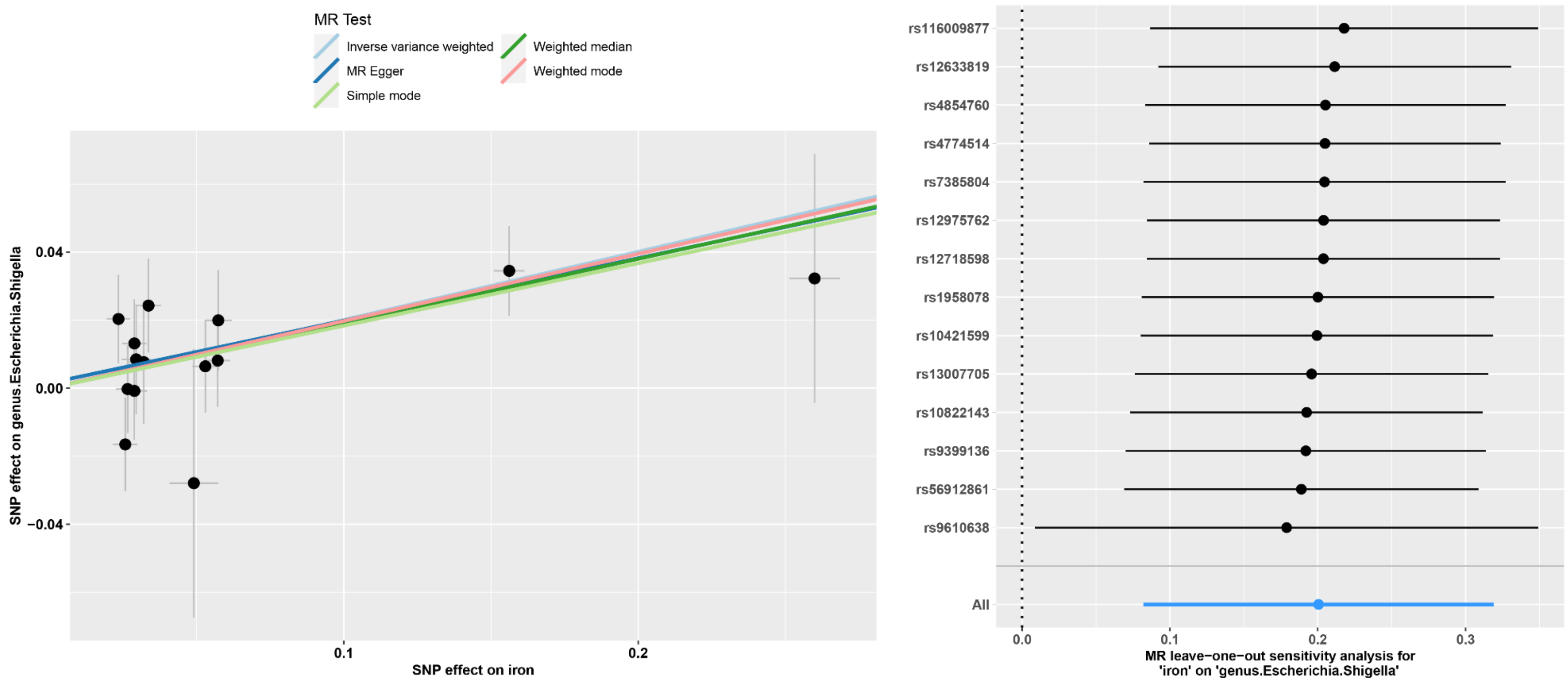

Figure S24. Genetic association of iron with genus *Terrisporobacter* (Scatter plot and Leave-one-out plot)

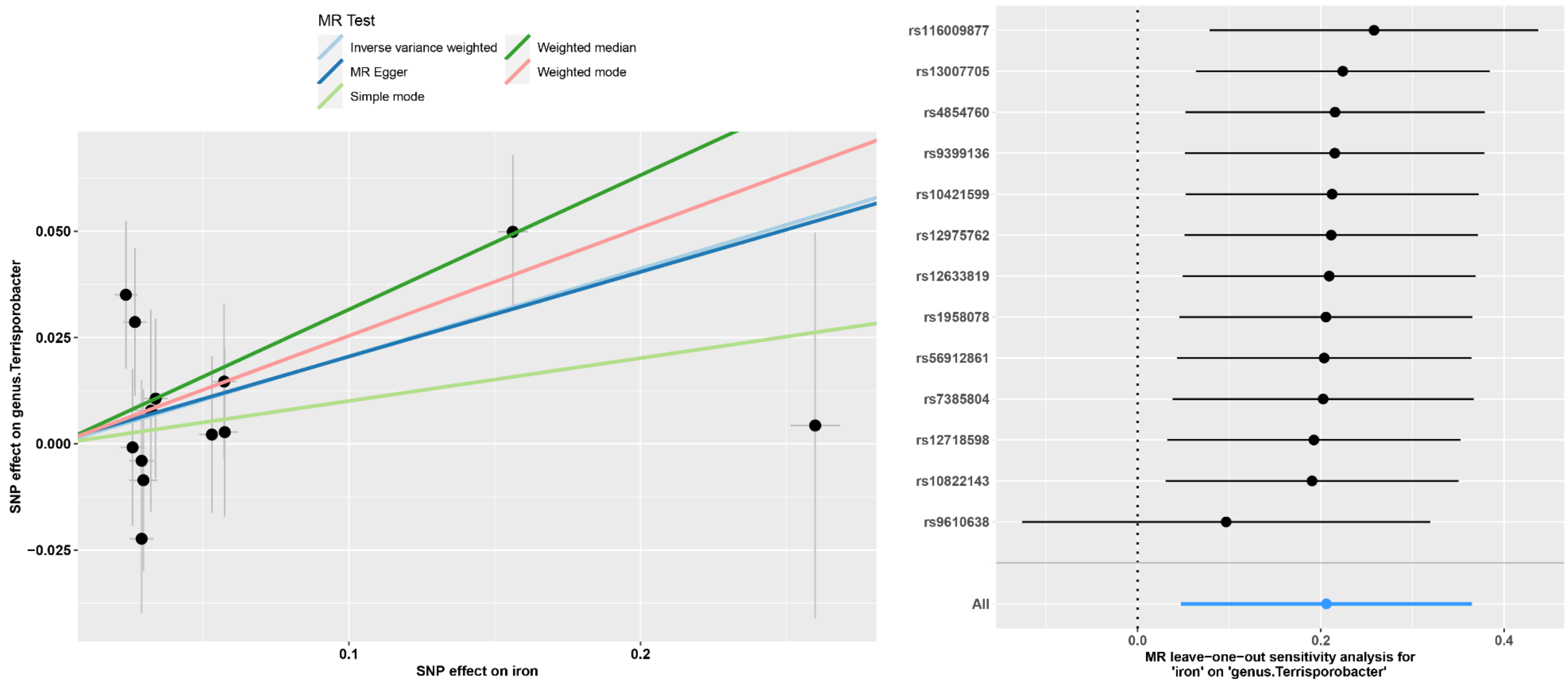

Figure S25. Genetic association of magnesium with class *Actinobacteria* (Scatter plot and Leave-one-out plot)

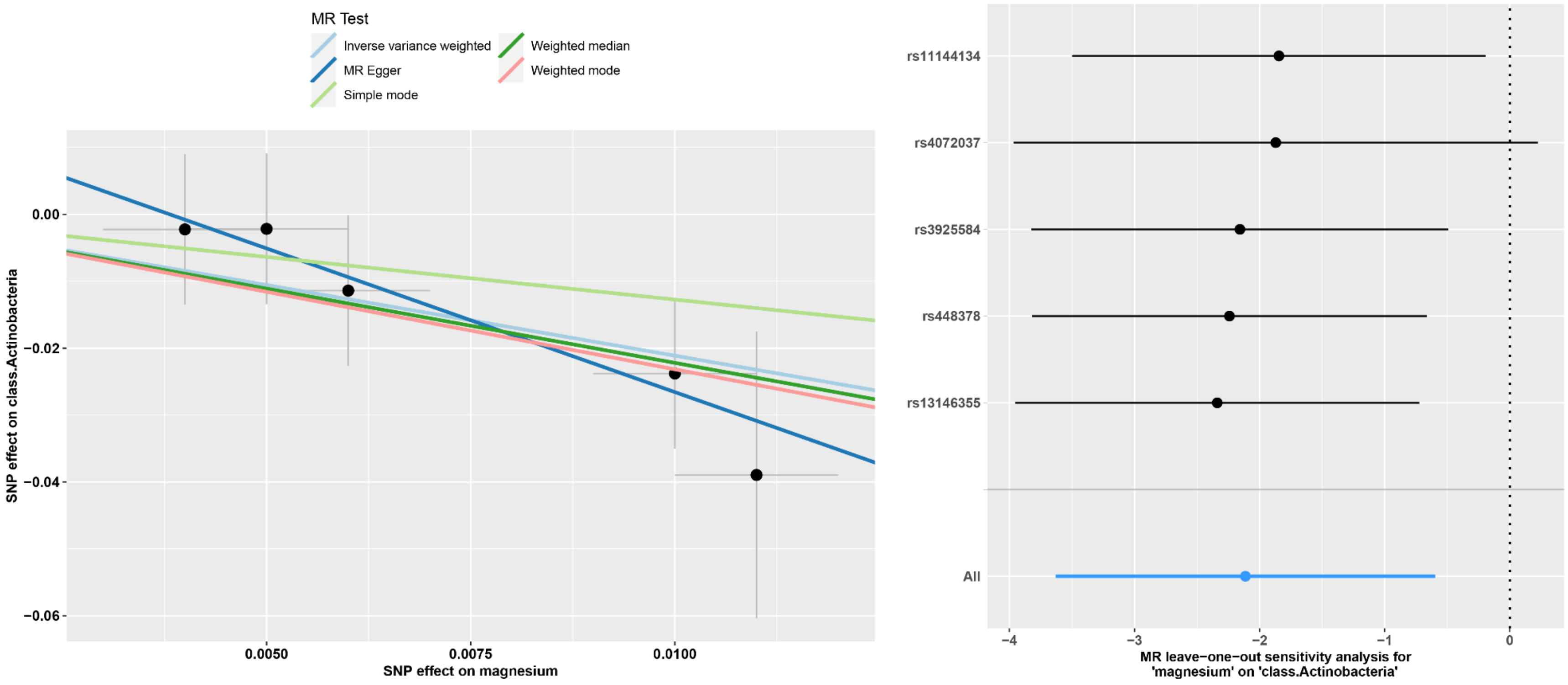

Figure S26. Genetic association of magnesium with order *Bifidobacteriales* (Scatter plot and Leave-one-out plot)

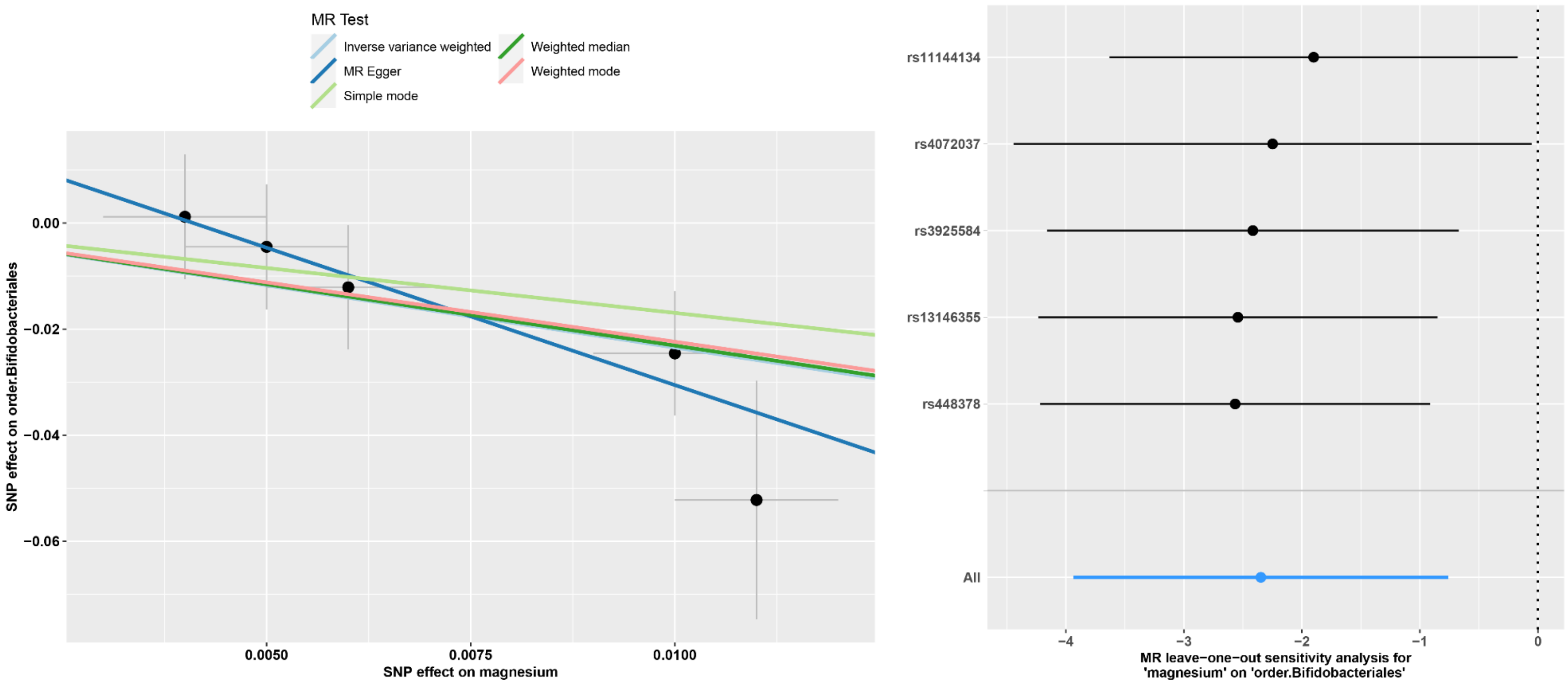

Figure S27. Genetic association of magnesium with family *Bifidobacteriaceae* (Scatter plot and Leave-one-out plot)

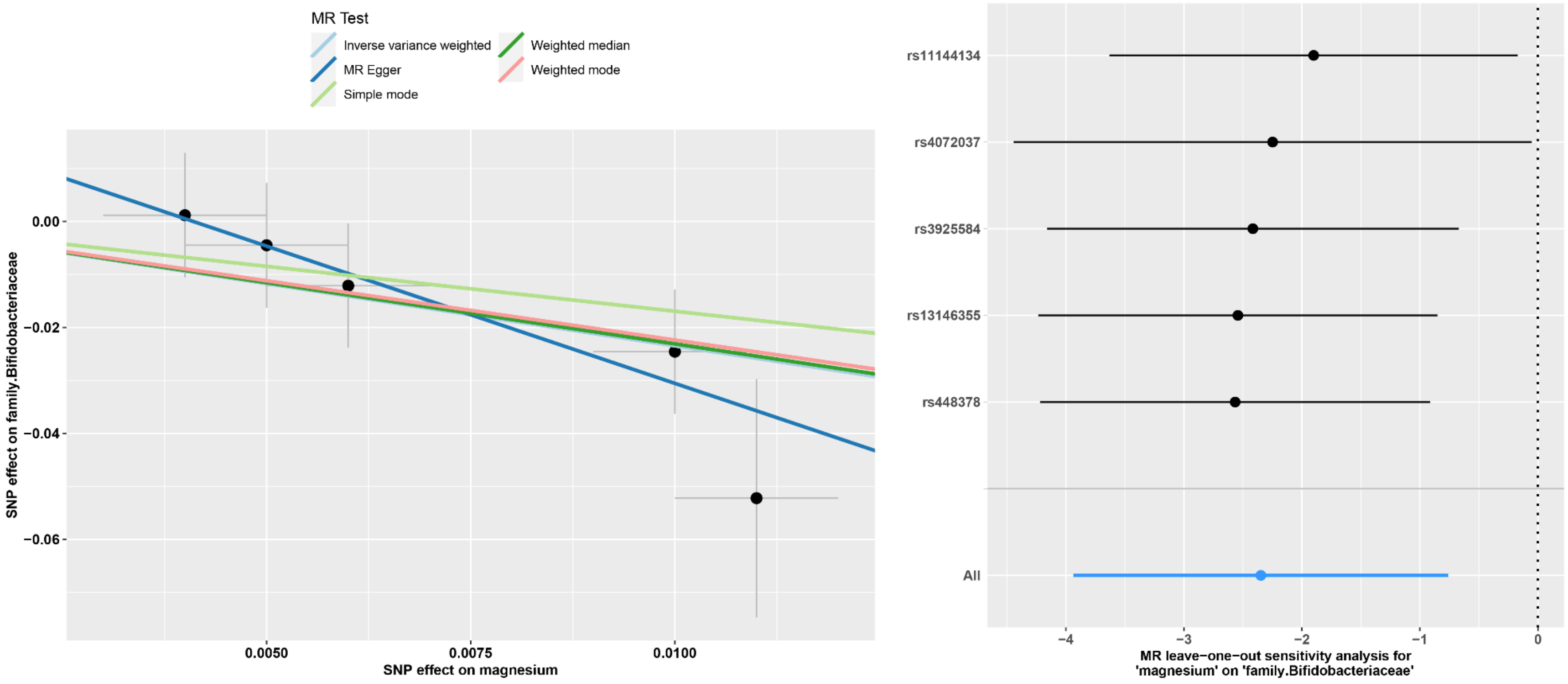

Figure S28. Genetic association of magnesium with genus *Bifidobacterium* (Scatter plot and Leave-one-out plot)

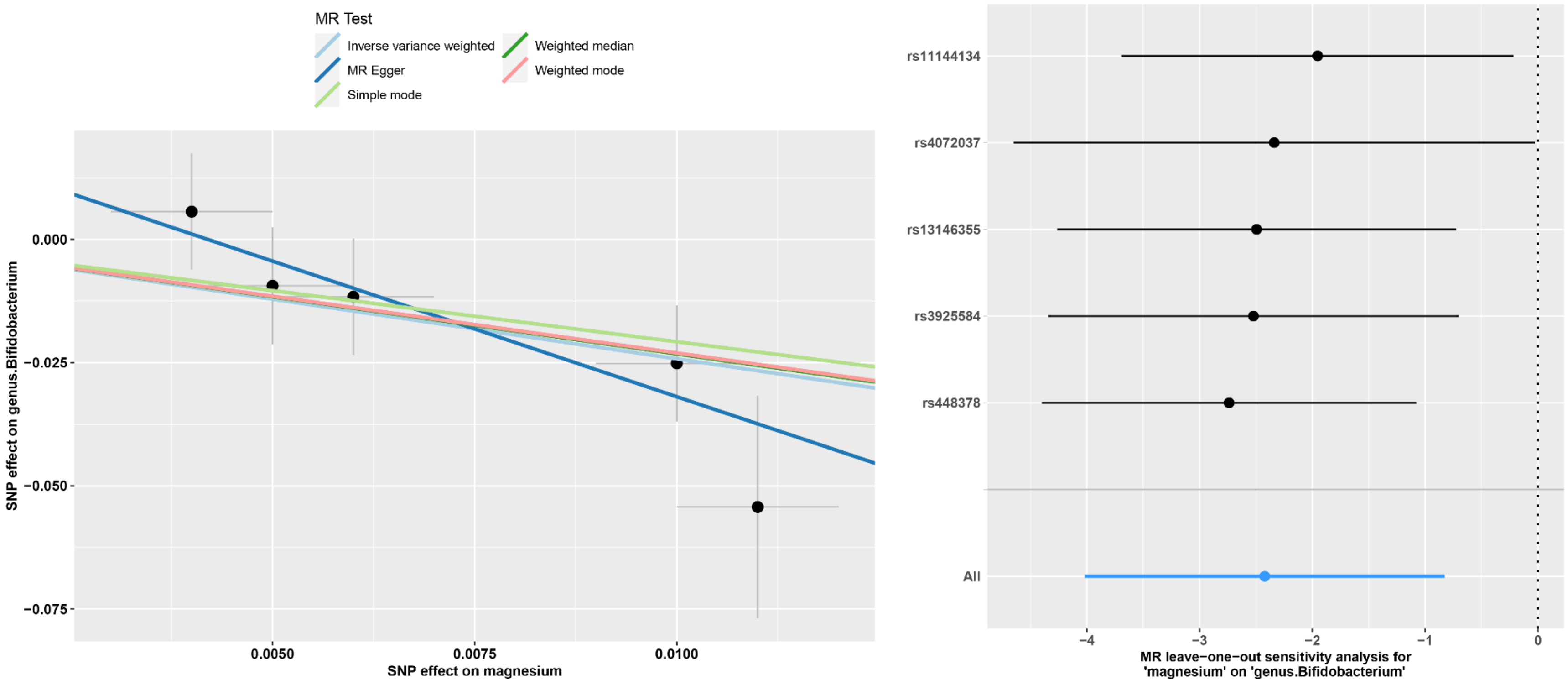

Figure S29. Genetic association of zinc with genus *Ruminococcustorquesgroup* (Scatter plot and Leave-one-out plot)

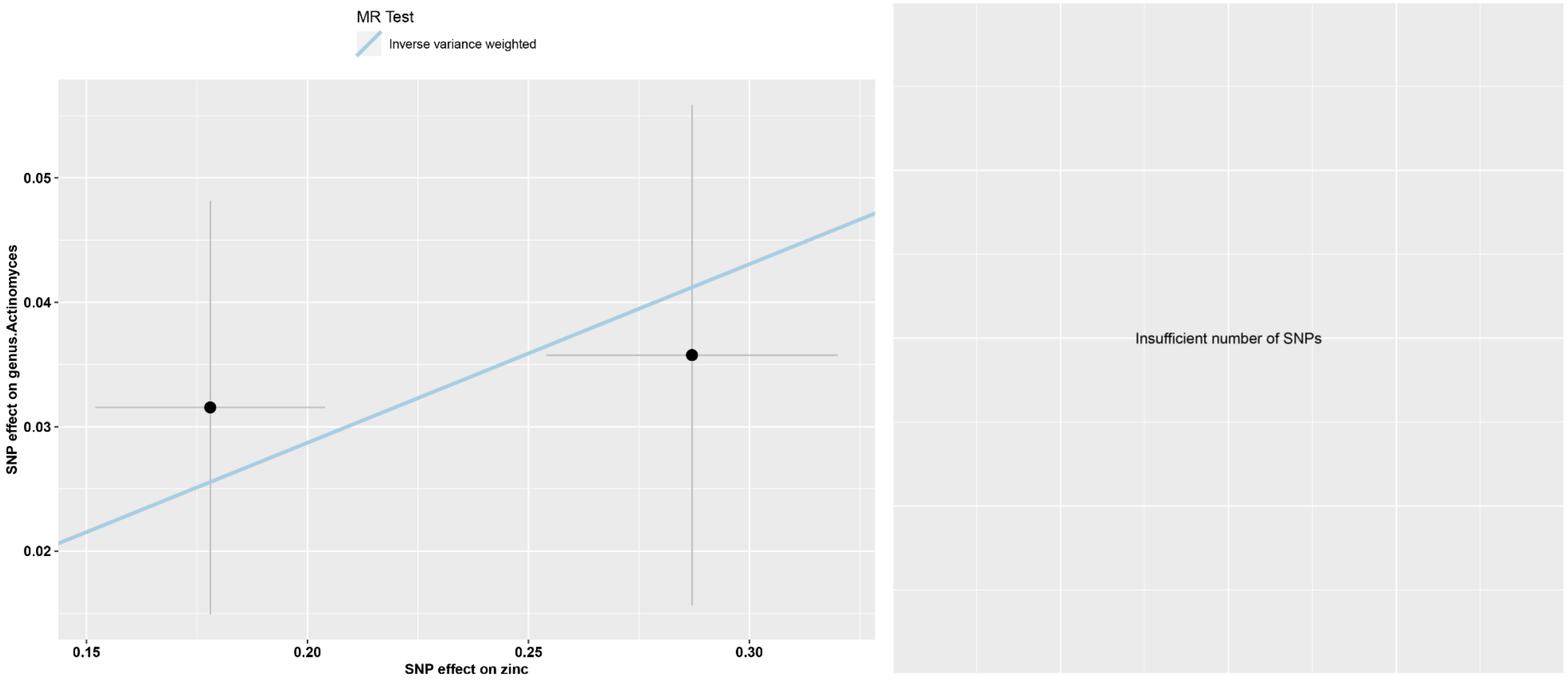

Figure S30. Genetic association of zinc with genus *Actinomyces* (Scatter plot and Leave-one-out plot)

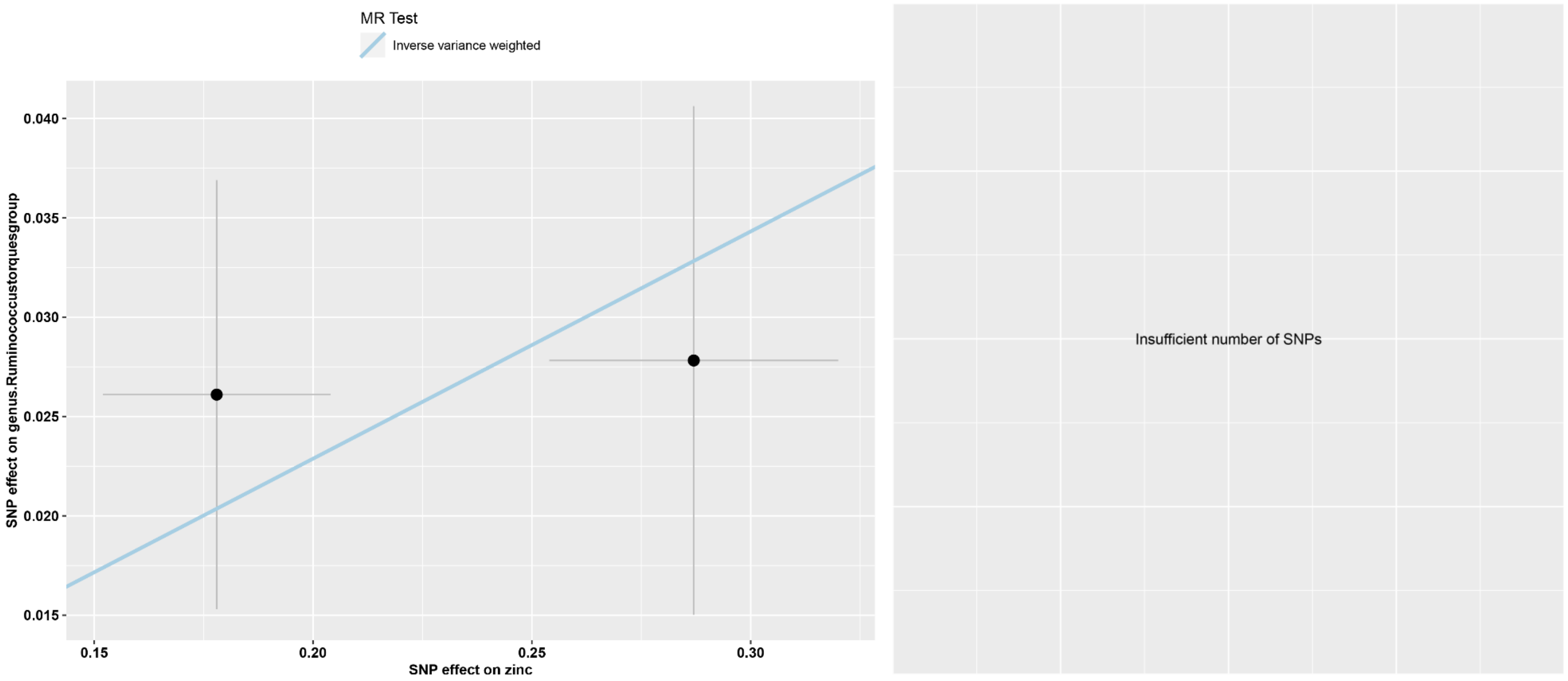

Supplement: Supplementary file 1 [file nutrients-16-00244-s001.zip › Additional File S2.pdf]
